# Growth factor gene *IGF1* is associated with bill size in the black-bellied seedcracker *Pyrenestes ostrinus*

**DOI:** 10.1038/s41467-018-07374-9

**Published:** 2018-11-19

**Authors:** Bridgett M. vonHoldt, Rebecca Y. Kartzinel, Christian D. Huber, Vinh Le Underwood, Ying Zhen, Kristen Ruegg, Kirk E. Lohmueller, Thomas B. Smith

**Affiliations:** 1 Ecology and Evolutionary Biology, Princeton University, Princeton, NJ 08544 USA; 2 Department of Ecology and Evolutionary Biology, Brown University, Providence, RI 02912 USA; 3 Ecology and Evolutionary Biology, University of California, Los Angeles, CA 90095 USA; 4 Center for Tropical Research, Institute for the Environment, University of California, Los Angeles, CA 90095 USA; 5 Department of Biology, Colorado State University, Fort Collins, CO 80523 USA

## Abstract

*Pyrenestes* finches are unique among birds in showing a non-sex-determined polymorphism in bill size and are considered a textbook example of disruptive selection. Morphs breed randomly with respect to bill size, and differ in diet and feeding performance relative to seed hardness. Previous breeding experiments are consistent with the polymorphism being controlled by a single genetic factor. Here, we use genome-wide pooled sequencing to explore the underlying genetic basis of bill morphology and identify a single candidate region. Targeted resequencing reveals extensive linkage disequilibrium across a 300 Kb region containing the insulin-like growth factor 1 (*IGF1*) gene, with a single 5-million-year-old haplotype associating with phenotypic dominance of the large-billed morph. We find no genetic similarities controlling bill size in the well-studied Darwin’s finches (*Geospiza*). Our results show how a single genetic factor may control bill size and provide a foundation for future studies to examine this phenomenon within and among avian species.

## Abstract (web summary)

Pyrenestes finches have a bill size polymorphism thought to be maintained by disruptive selection. Here, the authors identify a single candidate region, including insulin-like growth factor 1, differentiating small and large bill size morphs and a wider region differentiating the mega-billed morph.

## Introduction

How phenotypic variation arises and is maintained in natural populations is a fundamental question in evolutionary biology^1^. Despite the ubiquity of phenotypic polymorphisms in natural populations^2^, there are few instances in which the ecological and evolutionary factors that produce and maintain them are well understood, and still fewer where the genetic bases have been identified^3–5^. Resource polymorphisms, defined as intraspecific phenotypic variation associated with niche partitioning, can be under strong selection and are prime candidates for investigating the genetic, ecological, and evolutionary factors maintaining variation in natural populations and may represent important steps in speciation^6,7^.

**Fig. 1:**
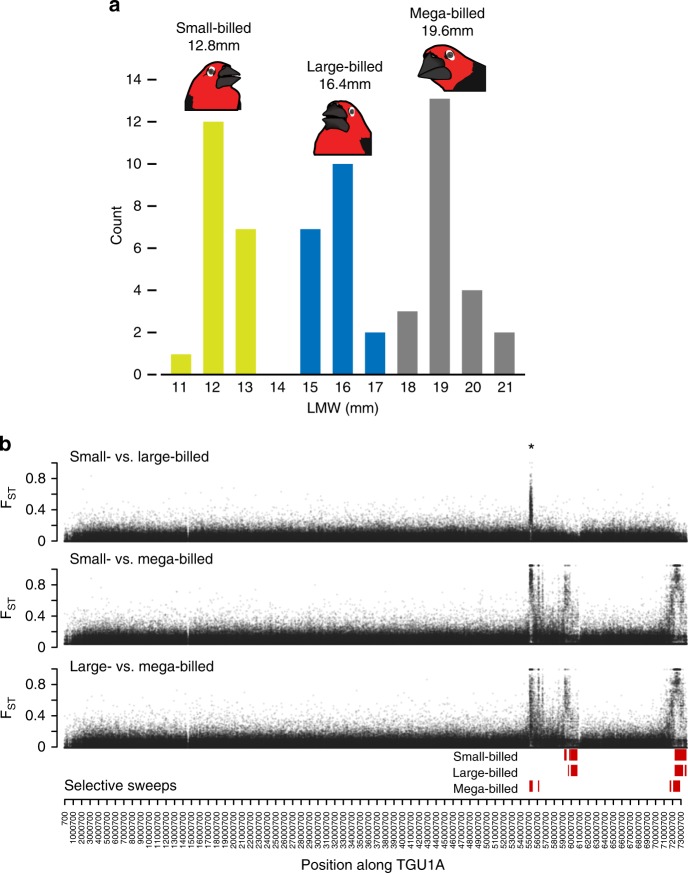
Variation in bill morphology and genetics in *Pyrenestes ostrinus*. **a** Lower mandible width (LMW) measurements identify three bill morphs (average LMW per morph is indicated) **b** Pairwise comparisons of genetic differentiation (*F*_ST_) among the three bill morphs along chromosome 1A (TGU1A) in 200 bp windows. Red bars indicate the genomic regions of predicted selective sweeps for each bill morph. The candidate region is denoted with an asterisk (*)

The black-bellied seedcracker (*Pyrenestes ostrinus*), a seed-eating estrildid finch common to the rainforests of Equatorial Africa, displays dramatic variation in bill size^8–11^ (Fig. 1a) and is a textbook example of disruptive selection maintaining a resource polymorphism in the wild^12–16^. Variation in bill size is discrete, extreme, and unrelated to sex or age, with three distinct size classes: small, large, and mega. When morphs co-occur within populations, individuals breed randomly with respect to bill size^8,17^. Morphs partition food resources during dry seasons when food is scarce^8,10,18^, with the small morphs preferring soft-seeded sedges (*Scleria goossensii*), and large and mega morphs specializing on increasingly hard-seeded species (*S. verrucosa* and *S. racemosa*, respectively). Disruptive selection affecting bill size primarily targets lower mandible width, which is related to the performance of cracking seeds of differing hardness^11,19^. Analysis of pedigreed crosses show that beak size differences in small and large morphs are due to a single, diallelic autosomal locus where the large-billed phenotype is dominant^11^. Beak size differences in small and large morphs have been shown to be due to differences in postnatal craniofacial development^20;^ however, beak size differences do not scale with body size^9^. In contrast, the transition to the mega phenotype involves concomitant increases in both beak and body size^9^, suggesting that the evolution of the mega phenotype involves more complex developmental processes that affect multiple traits^20^. The genetic basis of the mega morph is unknown.

Here, we use genome-wide pooled sequencing (Pool-seq) and targeted resequencing to investigate the genetic basis of this classic resource polymorphism. We identify a single candidate region underlying bill size differences in small and large morphs, confirming earlier work that suggested this trait is controlled by a single locus. In contrast, the mega morph is associated with more extensive variation on the same chromosome. Our results provide insights into how genetic variation contributes to discrete resource polymorphisms, and how they evolve and are maintained in natural populations.

## Results

### Identification of candidate regions underlying bill size

We performed genome-wide Pool-seq on 20 small-billed, 20 large-billed, and 21 mega-billed *P. ostrinus* morphs to identify candidate regions associated with bill morphology. We used pairwise comparisons among morphs to test two hypotheses: (1) that the differences between the small-billed and large-billed morphs are due to a single genetic locus, as predicted by breeding studies^11^, and^2^ that the mega-billed phenotype is the product of a series of stepwise mutations: from an ancestral small-billed phenotype, to a large-billed phenotype, and ultimately to mega-billed phenotype. To test these hypotheses, we obtained a minimum depth of 20-fold sequence coverage per morph (depth: small-billed = 20×, large-billed = 28×, mega-billed = 28×) from which we identified 6,262,908 SNPs genome-wide that were sequenced in at least two morphs and passed filters for allele frequency and *F*_ST_ estimation.

A sliding-window *F*_ST_ scan with 200 bp windows across the genome showed strong differentiation between small-billed and large-billed morphs in a single, approximately 300 Kb region on zebra finch chromosome 1A (hereafter referred to as TGU1A) (Fig. 1b). Of 14 total high-divergence (*F*_ST_ > 0.8) windows between small- and large-billed morphs, 13 are on TGU1A with 12 spanning the 300 Kb candidate region (Supplementary Table 1). Similarly, eight of nine fixed differences between the two morphs are also located in the same region of TGU1A (Supplementary Table 2). Because of the strong signal in a single contiguous region, and the expectation from a previous breeding study^11^ of Mendelian inheritance, we subsequently focused on this large region on TGU1A as the candidate region controlling bill size. To estimate the bounds of this candidate region, we identified individual SNPs with a large difference in allele frequency (Δ ≥ 0.9) between the small and large morphs. Over 60% of these sites (38 of 63 total SNPs) are concordant with the candidate region identified by the *F*_ST_ scan, spanning 301,630 bp (TGU1A: 55,070,008-55,371,638). The remaining highly divergent sites were distributed across other chromosomes (two on TGU2, six on TGU3, four on TGU4 and TGU12, and one each on TGU5, TGU6, TGU8, TGU15, TGU19, and TGUZ).

As with the comparison between the small-billed and large-billed morphs, TGU1A was enriched in high-*F*_ST_ windows in comparisons involving the mega-billed morph (Fig. 1B, Supplementary Table 1). Although the mega-billed individuals were collected from populations that were distinct from the small-billed and large-billed individuals, *F*_ST_ estimates indicated low levels of genetic structure among the three bill morphs. Thus, the differences observed on TGU1A cannot be attributable to population differentiation (median genome-wide *F*_ST_ with 200 bp sliding windows: small vs. large = 0.006; small vs. mega = 0.007; large vs. mega = 0.008) (Supplementary Fig. 1). Of 1483 high-divergence (*F*_ST_ > 0.8) genome-wide windows found in comparing the small-billed and mega-billed morphs, 1464 (99%) are on TGU1A. Similarly, 1088 of 1108 (98%) high-divergence windows between the large-billed and mega-billed morphs are on TGU1A. While small-billed morphs have significantly more high-divergence windows than large-billed morphs in comparisons with the mega-billed (Fisher’s exact test, *P* < 1.06 × 10^-13^), the high-divergence windows do not fully overlap between comparisons (978 windows shared between small and large in comparison with mega). We found similar patterns in the number of fixed differences between non-mega and mega-billed morphs, with nearly all fixed differences located on TGU1A (Supplementary Table 2). Further, we found significantly more fixed differences on TGU1A (Fisher’s exact test, *P* < 2.2 × 10^−16^) between the small- and mega-billed morphs (2270 of 2274 variants) than large- and mega-billed morphs (1647 of 1655 variants). However, only 1341 fixed differences are shared between the two comparisons (i.e., positions with alleles private to the mega morph).

While the comparison between the small-billed and mega-billed morph identified slightly more divergent sites than the large- and mega-billed comparison, the overall patterns of divergence between mega- and both non-mega-billed morphs were similar (Fig. 1b). In addition to strong divergence in the candidate region identified on TGU1A in the small- and large-billed morph comparison, the *F*_ST_ scan identified two additional, highly divergent regions downstream on the same chromosome (58,423,300-60,520,100 and 70,995,900-73,482,300).

We further investigated the 1341 SNPs carrying alleles private to the mega-billed morph to identify genes that may contribute to the mega phenotype. Of these, 392 private alleles are located within 28 annotated genes. Seven genes (*CCDC91*, *DDX11*, *IGF1*, *KLHL42*, *PTHLH*, *NUP37*, and *WASHC3*) are significantly enriched in private alleles (*P* *<* 0.05, bootstrap resampling with 10,000 replicates). We predicted effects of each genetic variant using *Ensembl*’s *Variant Effect Predictor* (VEP)^21^. Predictions on all 1341 mega-specific private alleles suggest that most are modifiers, which are either non-coding variants or variants that affect non-coding genes (moderate *n* = 3; modifier *n* = 481; low *n* = 5), with genes *CCDC91*, *DDX11*, *IGF1*, and *WASHC3* carrying the majority of modifier SNPs (*n* = 261, 34, 37, and 29, respectively) (Supplementary Table 3). We found no protein-coding variants.

### Estimating selective sweeps in the evolution of bill morphs

We used a hidden Markov model to estimate the posterior probability of selective sweeps on TGU1A for each bill morph. We identified five high-confidence sweep regions in each of the small-, large-, and mega-billed morphs with posterior probabilities of 1 (−log_10_(1−Prob) = inf) (Fig. 1B, Supplementary Data 1). All three morphs have a signal of selective sweeps at the telomeric end of TGU1A (>71.0 Mb). The mega-billed morph additionally contained a sweep concordant with the 300 Kb candidate region, while the small-billed and large-billed morphs carried sweeps in regions downstream of the candidate region (approximately 59.6–60.7 Mb).

### Genetic distinction between small-billed and large-billed morphs

**Fig. 2:**
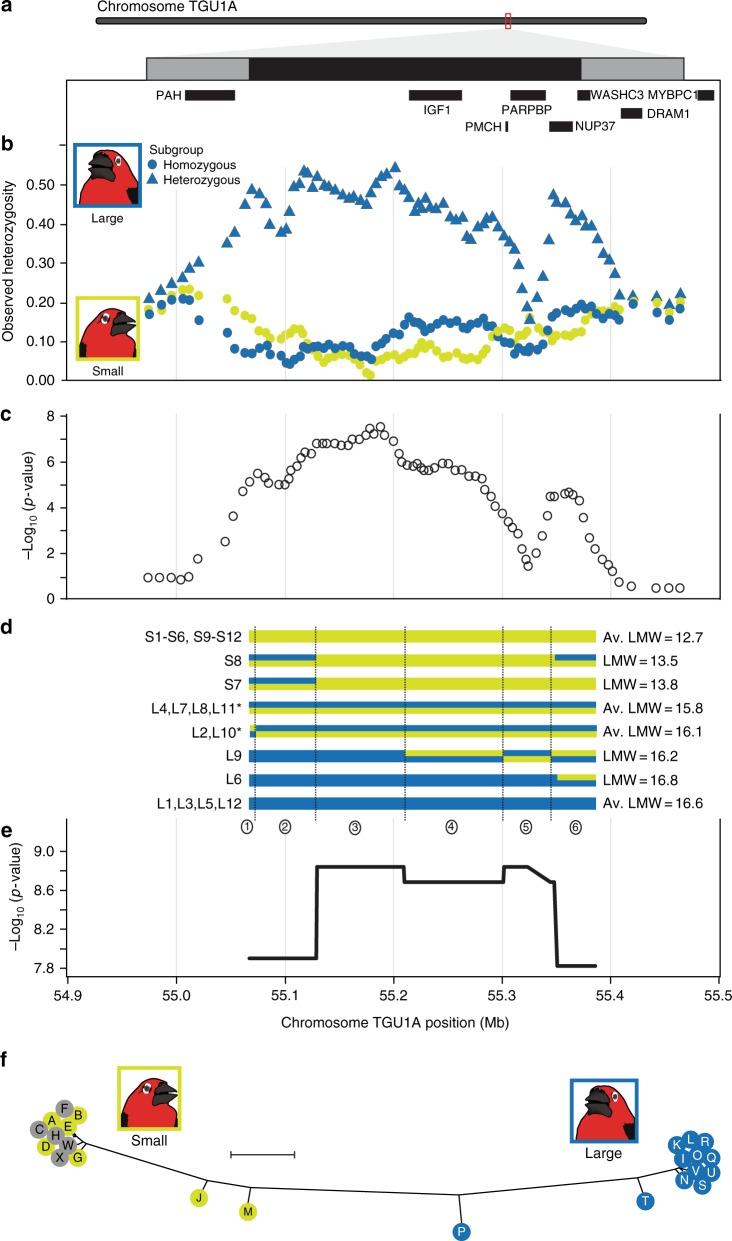
Zebra finch chromosome TGU1A and genetic variation associated with bill size. **a** The candidate and flanking region sequenced (TGU1A: 54,971,008–55,470,638). Annotated genes are indicated by black bars. A sliding window (200-SNP window, 100-SNP step) across 4717 SNPs depicts **b** observed heterozygosity (*H*_O_) by bill morphology (large-billed subgroups are defined in Fig. 3) and **c** Log-scaled significance of association between SNP variation and lower mandible width, a proxy for bill size, averaged over 200-SNP windows with a 100-SNP step. **d** Schematic of phased genotypes across the candidate region with associated lower mandible width (LMW, in millimeters; see Supplementary Table 8) where an asterisk (*) indicates large-billed individuals are members of the heterozygous subgroup, and **e** a quantitative association of haplotypes found within a region of high linkage disequilibrium with bill morphology. **f** A Neighbor joining haplotype tree for loci with high linkage disequilibrium. Haplotypes are color coded to the bill size. Gray shading indicates small-bill haplotypes that are heterozygous in large-billed individuals but are masked by a dominant large-bill haplotype (see Supplementary Table 8 for frequency details). The scale bar indicates a genetic distance of 0.1 units

**Fig. 3:**
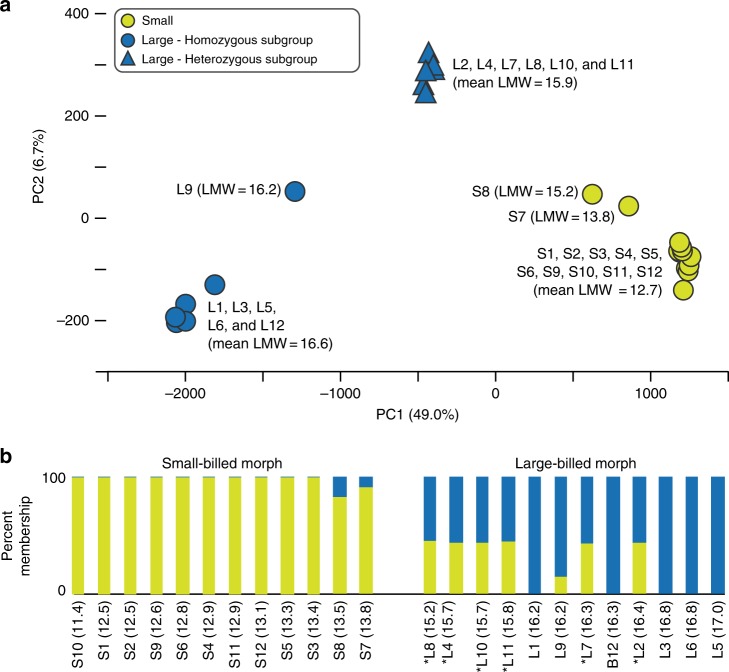
Cluster analysis of small- and large-billed *P. ostrinus*. **a** A principal component analysis of 4717 SNP genotypes across the candidate and flanking region, with two subgroups of the large-billed morph indicated. Lower mandible width (LMW in mm) and sample IDs are provided (see Supplementary Data 2). The proportion of variance explained by each component is provided along each axis. **b** Population structure analysis at *K* = 2 using 4717 SNPs from TGU1A (see Fig. S1 for *K* = 3–5). Sample IDs (LMW in parentheses) are along the *X*-axis, with birds ordered from smallest LMW (left) to largest LMW (right, in millimeters). The cross-validation value for *K* = 2 is *cv* = 0.462. Asterisks indicate that large-billed individuals are members of the heterozygous subgroup

To further investigate the genetic basis of the small-large polymorphism, we designed an array for targeted capture of the candidate region for deep resequencing (see Methods section). The targeted region contains the ~300 Kb candidate region, flanked by approximately 100 Kb on either side (TGU1A: 54,971,008-55,470,638). This region is gene-poor, with only 10 annotated genes (Fig. 2a). After filtering sites with more than 10% missing data, our enrichment assay captured 4717 SNPs with a median inter-SNP distance of 78 nucleotides on TGU1A from 12 small-billed and 12 large-billed *P. ostrinus*. A subset of 4122 SNPs are located within the candidate and flanking regions (Fig. 2a), with 1516 SNPs in eight genes (Supplementary Table 4). In a PCA of SNP genotypes, PC1 explains 49% of the variance and is strongly correlated with bill size (Pearson’s correlation coefficient, *r* = −0.933) (Fig. 3A) while PC2 explains 7% of the variance and is strongly correlated (*r* = 0.962) with individual observed heterozygosity. Individuals group into three clusters on the first two PCs: small-billed individuals with low heterozygosity (*n* = 12, mean *H*_O_ = 0.123, mean lower mandible width, LMW = 1.29 mm); large-billed individuals with low heterozygosity, hereafter referred to as the homozygous subgroup (*n* = 6, mean *H*_O_ = 0.132, mean LMW = 16.6 mm); and large-billed individuals with high heterozygosity, hereafter referred to as the heterozygous subgroup (*n* = 6, mean *H*_O_ = 0.366, mean LMW = 15.9 mm). The large-billed homozygous subgroup has marginally, but significantly larger bills than the heterozygous subgroup (one-tailed *t*-test *P* = 0.0067) (Fig. 3a). The large-billed heterozygous subgroup has significantly higher observed heterozygosity than both the homozygous subgroup and the small-billed individuals (*t*-test of unequal variance *P*-value: small-large_hom_ = 0.0242, small-large_het_ = 8.502 × 10^-258^, large_hom_-large_het_ = 1.458 × 10^-232^). Sliding-window analysis shows that the heterozygous large-billed subgroup individuals have consistently higher *H*_O_ through nearly the entire candidate region (Fig. 2b). Thus, this locus does appear to be dominant for the large-billed morph. However, we found evidence that this dominance may not be complete as the heterozygous large-billed subgroup has slightly smaller LMW than the homozygous large-billed subgroup.

The most strongly supported *Admixture*^22^ model predicts two distinct genetic clusters (*K* = 2) in small-billed and large-billed morphs within the candidate region (Fig. 3b, Supplementary Fig. 2), with a strong correspondence to bill morphology. Ten of 12 small-billed individuals are assigned entirely to a single cluster (*Q* > 0.99). The remaining two small-billed individuals (birds *S*7 and *S*8) are admixed but have majority membership in the small-billed cluster (*Q* values to the small-billed cluster: *S*7 = 0.91 and *S*8 = 0.83). These individuals also have the largest LMW of the small morphs. Mixed assignments were more common in the large morphs, with individuals from the heterozygous subgroup assigned 50% membership to each cluster, consistent with the high observed heterozygosity in these individuals. Thus, individuals cluster by phenotype when using SNPs from the candidate region.

We found that 1983 SNPs in the candidate region are significantly (*P* < 0.001) associated with bill size in a quantitative association analysis (Fig. 2c). SNP variation within three genes in the candidate region are significantly associated with LMW (median quantitative association *P*-values: *IGF1* = 6.45 × 10^−6^, *NUP37* = 1.46 × 10^−6^, and *WASHC3* = 1.58 × 10^−4^), and are candidate genes for influencing bill morphology (Supplementary Table 4).

### Haplotypes are strongly associated with bill morphs

After additional filtering for missing data, low MAF, and high *H*_O_, we assessed pairwise LD among 3989 SNPs located within in the candidate and flanking regions. We found that LD was highly variable, ranging from 0 to 0.999 (mean ± SD *r*^2^ = 0.111 ± 0.224) (Supplementary Fig. 3). Using *LDna*^23^, we identified 17 outlier clusters with strong LD among loci within a cluster and little LD between distant clusters (Supplementary Table 5). We focused on four of these outlier clusters, which had relatively large numbers of SNPs (between 90 and 373 each) that were in extremely high LD (mean *r*^2^ = 0.952–0.999) and additionally displayed characteristics of divergent haplotypes associated with bill size in small- and large-billed morphs. Namely, PCAs of each cluster clearly separated individuals into three genotypic groups along the first axis, which explains >99% of the total genetic variation at the component SNPs (Supplementary Fig. 4). Patterns of diversity within these genotypic groups are consistent with expectations for a diallelic locus. In every case, nearly all loci in the intermediate genotypic group are heterozygous, with nearly all loci homozygous in the two groups at either extreme of PC1 (Supplementary Fig. 4B). Genotypic groups within each of these four clusters are also significantly associated with LMW (ANOVA *P* < 1 × 10^-8^) (Supplementary Fig. 4C) and are consistent with representing small-billed and large-billed alleles.

These four outlier clusters are also in high LD with each other, with a high mean LD across all outlier-cluster SNPs (*r*^2^ mean = 0.770, range = 0.338–0.999). Given this strong LD, and that all four clusters are concordant in their genotypic patterns and association with phenotype, we pooled them into a single large cluster of 972 SNPs. This pooled cluster delineates a single extended region of high LD that spans 319.3 Kb (TGU1A: 55,067,422–55,386,717) and includes the entire candidate region (Supplementary Fig. 3). These 972 SNPs in high LD with each other are interspersed with 2180 SNPs showing little LD with each other, resulting in an overall lower level of LD within the outlier cluster boundaries (range *r*^2^ = 0.111–0.999) and are flanked by regions of lower LD (mean *r*^2^ ± SD = 0.015 ± 0.076, range = 0.000–0.999) (Supplementary Fig. 3).

We phased these 972 high-LD SNPs and identified 24 unique haplotypes that cluster into two divergent haplogroups associated with the small- or large-billed morphs (Fig. 2f, Supplementary Fig. 5; Supplementary Tables 6–8). The haplotypes most commonly associated with small- and large-billed morphs (haplotypes A and I, respectively) are 100% divergent at these SNP sites. There are twelve haplotypes that associated with the small-billed phenotype and have a low level of intra-haplogroup sequence divergence with an average of only 9% mismatches (Supplementary Tables 6, 7). Notably, haplotypes C, F, H, W, and X cluster within the small haplogroup but are found only as heterozygous genotypes within large-billed heterozygous subgroup. Based on sequence similarity, these haplotypes likely confer a small phenotype but are masked by the dominant action of the large haplotype in heterozygous individuals. The twelve haplotypes associated with the large-billed phenotype display a greater amount of haplotype diversity, also with 9% mismatches within the haplogroup (Supplementary Tables 6, 7). Six large-billed individuals are heterozygous for one large and one small haplotype, increasing the haplotype diversity within the large morphs to an average of 64% mismatches, and providing further support for the previous assertion of dominance for the large-bill phenotype^11^.

We found a significant quantitative association between number of copies of the small-billed haplotype A and LMW (*P* = 3.20 × 10^-7^, *ß* = −0.178 ± 0.02 s.e., *R*^2^ = 0.719, *T* = −7.34). A similar and inverse trend was noted with the large-billed haplotype I and LMW (*P* = 0.0153, *ß* = 0.146 ± 0.06 s.e., *R*^2^ = 0.249, *T* = 2.64). Further, we identified five recombination events on the phased haplotypes that produces six recombinant blocks in two small-billed and four large-billed individuals (birds S7, S8, L2, L6, L9, and L10) (Fig. 2d; Supplementary Fig. 6; Supplementary Table 8), all of which were significantly associated with LMW (*P* < 1.5 × 10^-9^). These recombination blocks align closely with the high LD SNP clusters identified in the *LDna* analysis (Fig. 2d, Supplementary Fig. 6; Supplementary Table 8). Finally of note are the two small-billed individuals with the largest LMW values for their phenotypic category (i.e., individuals S7 and S8), which were heterozygous for the large divergent allele along some portion of the candidate region (Fig. 2d).

### Noncoding variation in *IGF1* is associated with bill size

To identify genes impacted by phenotype-associated haplotypic variation, we used *VEP* to predict functional consequences of the 972 outlier-cluster SNPs within the candidate region. Of these, 966 SNPs were successfully annotated, with 418 predicted to affect annotated genes (Supplementary Table 9). Of the 418 total variants predicted to affect annotated genes, nearly half (204, or 49%) impact *IGF1*. These variants have predicted modifying effects, except for a single missense SNP with a predicted moderate impact (Supplementary Table 9). The remaining variants are distributed across *NUP37* (*n* = 67, 63 of which are modifiers), *PAH* (*n* = 65, all modifiers), *PARPBP* (*n* = 32, 31 of which are modifiers), *PMCH* (*n* = 23, 20 of which are modifiers), and *WASHC3* (*n* = 27, 26 of which are modifiers). *IGF1* and *PMCH* are significantly enriched in variants when accounting for gene size (Supplementary Table 10).

### Estimating the age of the bill-size associated haplotype

We used approximate Bayesian computation (ABC) to estimate the age of the large-billed haplotype. From the 200,000 simulated replicates, we derived a posterior distribution from the 200 simulations with the smallest Euclidean distance of summary statistics (nucleotide diversity within the ancestral, small-billed haplotype; nucleotide diversity within the derived, large-billed haplotype; and average divergence between the ancestral and derived haplotypes) to the data. A comparison of the prior distribution with the posterior distribution suggests that the summary statistics are highly informative about the equilibrium frequency and the age of the allele (Supplementary Fig. 7). The mode of the posterior distributions suggested an equilibrium frequency of 45% and an allele age of 2.21 × 4*N*_e_ generations. Further, we estimated a scaled mutation rate of 0.001 per base pair. We derived an effective population size of *N*_e_ = 114,000 individuals and an estimated age of the derived haplotype of 1 million generations or 5 million years old.

### Could the bill-associated haplotype be the result of an inversion?

The extensive, high linkage in the candidate region is suggestive of a chromosomal inversion. We hypothesized that an inversion fixed in large-billed and absent in small-billed birds would prevent recombination in individuals that are heterozygous for the inversion and lead to the high LD within the candidate region. Since an inversion mutation must have appeared on a single chromosome, and thus had no genetic variation when it first appeared, the ancestral and the derived (inverted) allele are not expected to share any polymorphisms. However, regions flanking an inversion are expected to experience free recombination with many shared polymorphisms between the ancestral and derived inversion background. We performed three analyses to determine whether the large-bill haplotype is the result of an inversion: (1) read-based analysis of genetic variation using paired-end sequence data to assess shared polymorphism and predict breakpoints; (2) PCR validation of predicted inversion breakpoints; and (3) sequence comparison of the small-billed and large-billed alleles. The read-based analysis showed that the candidate region is depleted of shared polymorphisms between the small- and large-billed haplotypes (Supplementary Fig. 8). This indicates reduced recombination within the candidate region and free recombination in flanking regions, which is consistent with predictions for an inversion. However, PCR validation of the left breakpoint (LBP) did not support the presence of an inversion in the large-bill haplotype (Supplementary Fig. 9).

Finally, to compare the sequences of two alleles, we assembled the large-bill allele from a single homozygous large-billed individual. The initial de novo assembly of the target region using the Illumina targeted resequencing data (20,523,728 paired-end and 6,610,292 single-end reads) produced 21,919 contigs, of which 1706 were greater than 1Kb in length. We additionally collected genome-wide, long-read sequence data from the same individual for scaffolding. Of 7.35 Gb total output, we retained 297 reads that spanned the target region (3,178,227 bp, or approximately 5.2x coverage). We used these long reads to scaffold all assembled contigs greater than 1 Kb. The final scaffold assembly consisted of 1688 contigs with a total length of 3,373,765 bp (N50 = 1762 bp) and was not improved by any combination of the alternative algorithms tested. We aligned all scaffolds greater than 5Kb against the candidate region in the zebra finch for a comparison with the small-billed allele. Four scaffolds, including the two largest in the assembly and with a total length of 488,349 bp, mapped successfully to the zebra finch reference (scaffold sizes in basepairs: 275,550; 147,188; 60,017; and 5594) and spanned the putative breakpoints predicted by the read-based genetic diversity analysis. In sum, sequence comparisons of the small- and large-billed alleles show no evidence of an inversion (Supplementary Fig. 10).

### How distinct is the mega-billed morph?

While small- and large-billed morphs differ in bill but not body size, the mega phenotype shows a dramatic increase in both bill and body size^20,24^. This may suggest that the evolution of the mega-billed phenotype followed a distinct genetic and developmental pathway from the large-billed phenotype^25^. However, the genetic basis of the mega morph has not previously been described. We collected sequence data for 21 mega-billed individuals and retained 5,411 SNPs within our targeted coordinates, with a median inter-SNP distance of 55 nucleotides. Genetic heterozygosity within the targeted region was significantly higher in the large-billed *P. ostrinus* compared to the small or mega-billed individuals (*H*_O_: small = 0.086, large = 0.195, mega = 0.094; one-tailed *t*-test of unequal variance *P*-value: small-large = 1.56 × 10^-131^, small-mega = 0.0155, large-mega = 10.7 × 10^-112^). In a PCA, the first axis explains 48.4% of the variation and separates individuals by beak morphology, with the small-billed and mega-billed morphs at the extremes and the large-billed morphs in the middle. The second PC (1.9% of variation explained) separates the large-billed morphs from the others, with the two large subgroups (homozygous and heterozygous) clustering separately (Supplementary Fig. 11). The topology of a NJ tree also supports the genetic distinctiveness of the mega-billed morph within the candidate region (Supplementary Fig. 12).

### Contrasting allelic variation with Darwin’s finches

We investigated the region homologous to the *P. ostrinus* candidate region in three species of Darwin’s finches (*Geospiza fuliginosa*, *G. fortis*, and *G. magnirostris*) to determine whether alleles associated with bill morphology in *P. ostrinus* were segregating or fixed within the highly-studied *Geospiza* system. We mapped sequence reads from 10 individuals across three *Geospiza* species and capture sequence reads from 12 small- and 12 large-billed *P. ostrinus* to the *G. fortis* reference genome (Supplementary Table 11). We retained 10,131 SNPs with a minimum of 10x sequence coverage across 59 scaffolds (Supplementary Table 11). Of the scaffolds with mapped data, 98% of the sites (*n* = 9911) mapped to scaffold 10, which we infer to be the homologous region to the candidate region. We restricted our survey to scaffold 10. Of these sites, after excluding three *fuliginosa* and three *magnirostris* individuals with missing data > 50% on scaffold 10, missing data was highest for *G. magnirostris* (Mean proportion missing: *P. ostrinus*: small = 0.00, large = 0.00; *Geospiza*: *fortis* = 0.34, *fuliginosa* = 0.34, *magnirostris* = 0.42). A PCA of sites mapping to *G. fortis* scaffold 10 revealed substantial genotypic divergence between *P. ostrinus* and *Geospiza* species, suggesting a divergent underlying genetic architecture determining bill morphology (Supplementary Fig.13A). Further, a PCA of 744 sites that differentiated *P. ostrinus* small- and large-billed individuals (*F*_ST_ > 0.5) shows all *Geospiza* species clustering together, separate from the large- and small-billed *P. ostrinus* (Supplementary Fig. 13B).

## Discussion

We provide a genome-level view of the mechanisms underlying a bill size polymorphism in the black-bellied seedcracker (*Pyrenestes ostrinus*). Our results are remarkably consistent with an earlier pedigree analysis^11^ that suggested a dominant large-billed allele at a single locus controls bill size differences between small and large morphs. We identify this locus as a 300Kb gene-poor region of low recombination on TGU1A (e.g., refs. ^26–32^). We further present evidence that insulin-like growth factor 1 (*IGF1*), which falls within this 300 Kb candidate region, is likely important in determining bill size differences between small- and large-billed morphs. In contrast, our analysis suggests that the third, mega-billed morph is the result of a more expansive set of genetic changes spanning a larger region on TGU1A.

Using high-throughput sequencing of small- and large-billed individuals, we identified a single 300 Kb gene-poor region on chromosome TGU1A segregating with bill morphology. Targeted resequencing revealed extensive linkage and strong divergence between haplotypes associated with the small and large morphs. Both haplotypes segregate within the population, and as expected, the genotypic patterns indicate that the large-billed allele is dominant to the small-billed allele. However, dominance does not appear to be complete, as heterozygous large individuals (carrying one large and one small allele) are on average smaller than the homozygous large-billed individuals. Additionally, individuals carrying recombinant haplotypes often scale in bill size according to the amount of the large haplotype carried. For example, the candidate region in recombinant individuals S7 and S8 contains 17 and 9% large haplotype, respectively, and these individuals have the largest bills of the small morphs. This suggests perhaps that there are multiple variants throughout the candidate region affecting bill size in *P. ostrinus*. By inspecting patterns of recombination in the small-billed S7 and S8, which are only heterozygous at the edges of the candidate region, the variant(s) driving the transition from small to large morph should fall in recombination blocks 3, 4, or 5 (TGU1A: 55,130,138–55,324,218). The annotated genes in this region are *IGF1*, *PMCH*, and *PARPBP*. Due to the lack of known functional relevance of *PMCH* and *PARPBP* (encoding a preproprotein and DNA repair functions, respectively) and a paucity of modifying variants associated with them, we focus our discussion on *IGF1*.

The persistence of these divergent haplotypes with extensive LD is likely due to the region’s low recombination rate, which appears to be phylogenetically conserved^26–32^. We further used a coalescence approach to estimate the molecular age of the derived haplotype associated with the large-billed morphology to be at least 5 million years old. One possible mechanism that could maintain the observed extensive LD is a chromosomal inversion, which suppresses local recombination (e.g., ref. ^33^). Chromosomal inversions have been identified as responsible for phenotypic variants in some taxa (e.g. mimicry in butterflies^34^; rose-comb in chickens^35^; colony organization in ants^36^; plumage and behavior in ruffs^37^). We collected long-read sequence data and surveyed the candidate region to identify possible breakpoints and a signal of genetic variation that might indicate a possible inversion, with subsequent PCR amplification to validate potential breakpoints. We found no evidence for an inversion underlying the bill size polymorphism. Although we cannot definitively reject the hypothesis that there is an inversion, other possible mechanisms (e.g., incomplete lineage sorting of ancestral haplotypes or possible introgression) could explain the origin of the large-bill haplotype.

Of the variants characterizing the extended haplotypes associated with bill morphology, 43% are non-coding and predicted to be modifiers, with the rest falling in intergenic regions. Nearly half of these modifying variants are located upstream of *IGF1*, which has a well-known role in body size scaling in many taxa (e.g. chickens^38^; chinook salmon^39^; tilapia^40^; domestic dogs^41^; brown house snakes^42^; cattle^43^) and skeletal development (reviewed in ref. ^44^). Further, *IGF1* is regulated by multiple genes and their proteins and encodes a highly flexible pleiotropic secretory polypeptide hormone^45–47^. Thus, it would not be surprising for *IGF1*’s regulatory elements to play important roles in shaping craniofacial morphology and scaling.

Changes in *IGF1* activity are known to be responsible for dramatic changes in size and shape of target tissues (e.g., refs. ^48,49^). For example, in domestic dogs a single haplotype of *IGF1*’s promoter is associated with small body size and is nearly absent in larger breeds^41^. Of course, dog breeds are maintained via selective breeding, which is not the case in *P. ostrinus*, where the bill size polymorphism follows Mendelian segregation within randomly mating populations. In the case of *P. ostrinus*, the association of genetic variation at *IGF1* resides in a region of phylogenetically conserved low recombination rates, which maintains a high level of LD within the haplotype associated with bill size.

To our knowledge this is first study to suggest that *IGF1* plays a major role in determining avian bill morphology. Genetic mapping studies of Darwin’s finches (*Geospiza*) and great tits (*Parus major*) have associated genetic and regulatory variation of genes with known roles in craniofacial morphology to bill size and shape^48–54^, but have not implicated *IGF1*. While niche partitioning of food resources has driven the evolution of bill morphology in both *Geospiza* and *P. ostrinus*, there are critical differences between these two systems. Most obvious is that morphological variation in bill size and shape in *Geospiza* typically distinguish different species^55,56^ with restricted gene flow; in contrast, the bill polymorphism in *P. ostrinus* is maintained within randomly-mating populations^8,17^. The single locus controlling bill size in small and large morphs of *P. ostrinus* contrasts with the apparent polygenic control of bill size and shape that characterizes interspecific variation in *Geospiza*. To more fully assess whether our candidate region in *P. ostrinus* also contributes to bill phenotypic variation in *Geospiza*, we compared allelic variation in our candidate region with the homologous region in several *Geospiza* species. We find that the alleles associated with *P. ostrinus* bill variation do not segregate with *Geospiza* bill morphologies.

In contrast to the small-billed and large-billed morphs that display Mendelian inheritance, we find that the mega-billed morph is under more complex control. The mega-billed morphs occur both geographically separate and together with the other bill morphs. All morphs interbreed where they are found together^57,58^. Megas are distributed only in drier ecotone regions that characterize the transition between rainforest and savanna, where the extremely hard seeds of the sedge *Scleria racemosa* occur. We found evidence of selective sweeps in regions of TGU1A that are strongly divergent between the mega-billed and non-mega-billed morphs, consistent with positive resource-based selection driving the evolution of the mega-billed morph, likely due to the abundance of *S. racemosa*. In contrast, we do not find a signature of selective sweeps in the candidate region for the small- and large-billed morphs, which are subject to disruptive selection where both soft (*S. goossensii*) and hard (*S. verrucosa*) seeds occur and the extremely hard seeded *S. racemosa* is absent. In megas, we identified additional variants downstream of the candidate region, suggesting that the genetic basis for this extreme phenotype is more complex than in small and large morphs. Although mega-bills were sampled from multiple populations, we found very low levels of genome-wide genetic divergence (e.g., *F*_ST_ < 0.01) that indicates geographic population structure is unlikely a contributing factor to bill differences.

We have shown that bill size differences between small- and large-billed morphs of *P. ostrinus*, which differ in bill size but little in body size, have a simple genetic basis likely involving differential regulation of *IGF1*. The evolutionary transition to the mega-billed morph, which is larger in both bill and body size, involves changes across a more extensive region on the same chromosome but does not seem to have resulted from a simple stepwise evolution from the large-bill morph. Additional research will be necessary to more fully understand the genetic basis of the bill size polymorphism in *P. ostrinus*, especially with respect to the mega-billed morph. Further, given the importance of *IGF1* and its role in biological scaling, regulatory and transcriptional variation would be a fruitful area of future work to explore the specific evolution of scaling. Differences among related bird species are frequently characterized by beak size, a trait typically highly correlated with feeding, performance and adaptive divergence among populations and species^59^. Thus, the results of this research underline the importance of future work to understand the role of *IGF1* in the evolution of avian bill size and the diversification of birds more broadly.

## Methods

### Pool-seq scan for associations with bill size

We performed Pool-seq to identify candidate regions associated with bill size in all three morphs. By barcoding the phenotype rather than the individual, Pool-seq is a cost-effective method for estimating allele frequencies. We prepared genomic DNA using QIAamp DNA mini kits (Qiagen) from 20 small-billed and 20 large-billed adult *P. ostrinus* individuals captured between 1986 and 2007 from the same population in Ndibi, Cameroon and from 21 mega-billed individuals from four different populations in Cameroon^58^ (Supplementary Data 2). While the mega-billed individuals were sampled from different populations from that of the small-billed and large-billed individuals (Supplementary Data 2),, all three bill morphs are found in one of the populations (Tibati; Supplementary Data 2), where they are known to interbreed. Individuals were classified into the three bill-size phenotypic categories based on their lower mandible width (LMW) as a continuous quantitative trait (mean LMW: small = 12.8 mm, large = 16.4 mm, mega = 19.6 mm)^11,57^. DNA was quantified using a Qubit 2.0 Fluorometer and checked on a 2% agarose gel for degradation. We constructed Pool-seq libraries for paired-end sequencing (2 × 100 nt) on an Illumina HiSeq 4000 using the TruSeq DNA PCR-Free LT kit Set A (Illumina), tagging each bill morph with a unique barcode. Briefly, DNA was fragmented with a Bioruptor NGS sonicator (Diagenode) to an average insert size of 500 bp (5 cycles of 30 s on and 90 s off on the high setting), ends were repaired with adenine on the 3′ ends, followed by ligation of dual adapters, and selection of 300 bp fragments with Agencourt AMPure XP beads (Beckman Coulter). Library quality was assessed with an Agilent 2100 BioAnalyzer (Agilent) and concentrations were estimated with a Qubit 2.0 Fluorometer. Libraries were then standardized to 10 nM, and all libraries were pooled into two lanes at UC Berkeley Genomics Core.

For demultiplexing, we retained sequences with no more than two mismatches to that of the expected index. All reads were trimmed using CLC Genomics Workbench, using the Illumina TruSeq adapter sequences searched on both strands and removing reads that were < 20 bases and of low quality (score < 0.05; removing a maximum of 2 ambiguous nucleotides). Reads were mapped to the zebra finch reference genome (taeGut2) using default parameters in *stampy*^60^. *Stampy* is useful for mapping reads from a species that is highly divergent to a reference genome^60^. We used *PoPoolation2*^61^ to calculate differences in allele frequency (module SNP-frequency-diff.pl) and pairwise genetic differentiation (module fst-sliding.pl) among the three morphs. Allele frequency differences were called only for sites with a minimum minor allele depth of 2, an overall minimum depth of 10, and a maximum depth of 40. *F*_ST_ estimation was in 200 bp windows with a 200 bp step size with the same coverage requirements and discarding windows with insufficient coverage along their entire length. We additionally used the *mpileup* function in *SAMtools*^62,63^ for variant calling, filtering to retain only bi-allelic SNPs with a minimum depth of 10x and a minimum mapping quality score of 20, and excluding SNPs located within predicted indels. We identified SNPs with a difference in allele frequency between small and large morphs greater than 0.9 to establish the bounds of the candidate region.

To identify potential candidate genes underlying the mega-billed phenotype, we identified SNPs with fixed differences between mega and non-mega (small-billed and large-billed) morphs (i.e., loci with alleles private to the mega morph). We assessed the putative effects of the mega-private alleles on gene function with Ensembl’s *Variant Effect Predictor* (VEP)^21^. To identify genes that carry an excess of mega-billed private alleles relative to random expectation, we performed a bootstrap resampling with 10,000 replicates. Each bootstrap replicate consisted of random draws with replacement of genes from all genes represented in the dataset, with the probability of selecting each gene weighted by its total length. This yielded a null distribution of the number of expected mega-billed private alleles for each gene. *P*-values were calculated per gene as the proportion of null replicates that were greater than the number of observed mega-private alleles.

We additionally estimated the posterior probability of selective sweeps in each bill morph on TGU1A with *Pool-hmm*^64^, which uses patterns of allele frequencies in Pool-seq data to predict the most likely of three possible states (neutral, intermediate, or selection) for each polymorphic site. We analyzed each morph separately, with theta (-*t*) set at the default of 0.005 and the per-site probability of transition among states (-*k*) at 10^-8^. We applied a strict threshold of a reported posterior probability of *inf*, reflecting the lowest *P*-value detectable with this method, to identify high-confidence selective sweep regions.

### Targeted resequencing of the candidate region

The window-based *F*_ST_ scan identified a single region of high divergence on TGU1A that putatively underlies bill size differences between small and large morphs (see Results section). To refine the bounds of this candidate region, we used a heuristic metric for identifying divergent allele frequencies relative to the phenotype, referred to here as a delta value (Δ). Delta values were estimated as the absolute difference of the reference allele frequency between the small-billed and large-billed *P. ostrinus*. To identify highly divergent SNPs, we filtered to retain SNPs with high delta values (Δ ≥ 0.9) for a more conservative threshold.

We identified a single ~300 Kb candidate region on chromosome TGU1A (hereafter the candidate region) that was enriched with highly divergent alleles between small and large morphs. To obtain individual-level genotypic data to discover individual variants associated with bill morphology, we designed baits to target and enrich genomic libraries for this candidate region. Due to the known Mendelian inheritance for small- and large-billed morphs, we selected a subset of 12 large- and 12 small-billed individuals from the Pool-seq samples for targeted enrichment using 80mer baits designed and conducted by MYcroarray©. The target region contains the 301,630 bp candidate region, and is additionally flanked on both sides by ~100 Kb for a total enrichment of 499,630 bp (TGU1A: 54,971,008–55,470,638 in taeGut2) (Fig. 2a). MYcroarray prepared libraries with an average insert size of ~300 bases, individuals were tagged with dual-index barcodes and pooled into a single lane for single-end 1 × 67 nt sequencing and an additional lane for paired-end 2 × 67 nt sequencing on an Illumina HiSeq2500. For demultiplexing, we retained sequences with no mismatches to the expected index. All reads were trimmed using *cutadapt*^65^ with the same parameters as implemented in CLC (described above) and mapped to the reference zebra finch genome using *stampy*. PCR duplicates were removed using *Picard* tools (http://broadinstitute.github.io/picard). We used *ANGSD*^66^ to call SNP genotypes with a minimum depth of 30-fold sequence coverage, a minimum mapping quality of 20, and a minimum variant quality of 60. All downstream analysis contained only the SNPs and sequence that mapped within the coordinates of the candidate and flanking regions in taeGut2. We used gene annotations from taeGut2 downloaded from Ensembl BioMart^67^ to annotate each SNP.

We estimated observed heterozygosity (*H*_O_) for the resequenced 12 small- and 12 large-billed morphs using *PLINK* v1.9^68^, implementing a sliding window approach (window size = 200 SNPs, 100-SNP step) to visualize the mean values per window. A principal component analysis (PCA) was conducted using *flashPCA*^69^ on SNP genotypes. We analyzed the SNP genotypes for genetic structure with the program *Admixture*^22^, using the --cv flag for a cross-validation evaluation of the fit of each genetic partition (*K*). We used *PLINK* to associate genetic variation with the quantitative trait LMW. This method estimates the regression coefficient (ß) and the significance value from the Wald Test, also called a Wald Chi-Squared test, which tests if ß at a site is non-zero.

### Analysis of linkage disequilibrium in the candidate region

To investigate patterns of LD within the candidate region associated with bill morphology, we calculated pairwise LD among SNPs from the targeted resequencing data. SNPs were excluded from LD analysis if a site had > 10% missing data, high observed heterozygosity, (*H*_O_ > 50%), and a low minor allele frequency (MAF < 20%). We estimated pairwise LD for SNPs within the candidate region as the correlation coefficient *r*^2^ using the LD function in the R package *genetics*^70^. Patterns of LD revealed that SNPs are a mixture of strongly correlated sites interspersed with sites that appear statistically independent (see Results). To extract and characterize these strongly correlated sites, we used a network analytic approach implemented in *LDna*^23^. By treating network vertices as loci, and edges connecting vertices as LD between pairs of loci, *LDna* identifies clusters of SNPs that are in stronger LD with each other than they are to the rest of the network, and that cannot be subdivided into smaller groups. Cluster delimitation in *LDna* is controlled by two user-defined parameters: |*E*|_min_, the minimum number of edges required above an LD threshold; and *ϕ*, a constant that defines the stringency for considering a cluster to be a statistical outlier. Following the recommendation by Kemppainen et al. (ref. ^23^), we tested |*E*|_*min*_ values between 1 and 10% of the total number of loci, and tested *ϕ* values ranging from 3 (least stringent) to 7 (most stringent). Outlier clusters (groups of SNPs that are exceptionally large and/or in exceptionally strong LD) are referred to as single-outlier clusters in *LDna* terminology; for simplicity, we refer to them as outlier clusters.

### Haplotype phasing and functional annotation

To assess haplotype structure across the candidate region to determine if a single haplotype carries a candidate variant associated with bill morphology, we used *SHAPEIT*^71^ to phase the same SNP set analyzed by *LDna* and a reduced recombination rate of 0.1 cM/Mb for phasing due to previous findings that this region has a phylogenetically conserved low rate of recombination (e.g., refs. ^26–32^). The resulting phased haplotypes were polarized based on the number of haplotypes carried that were derived from the large haplogroup. We used these haplotype counts (0, 1, or 2) per phased site as a multi-locus diploid genotype to conduct a quantitative association test in *PLINK* across the candidate region. To explore relationships among haplotypes, we constructed and viewed two types of unrooted haplotype trees, neighbor joining and median network joining trees, in *SplitsTree* using default parameters^72^. We additionally used VEP to annotate the inferred functional and phenotypic impact of SNP variants. To identify genes that carried an excess of variants while controlling for gene size, we performed a bootstrap resampling as above: 10,000 replicates of random draws of each gene, with genes weighted by their total length.

### Estimating the age of the large-bill haplotype

The high level of divergence between the large-bill and small-bill haplotypes seems incompatible with a neutral model and suggests that the derived haplotype associated with the dominantly inherited large-billed morphology is old and under selection. We estimated the age of the derived haplotype assuming long-term balancing selection. To this end, we first simulated 200,000 replicates of a heterozygous advantage model where trajectories of a selected allele start with a single copy and over time converge to an equilibrium frequency defined by the fitness of each genotype. The simulated trajectories have three parameters: (1) the equilibrium frequency, (2) the heterozygous fitness advantage, and (3) the age of the allele. We randomly sampled these parameters from uniform prior distributions with a range of 50–1000 for the scaled heterozygous selection coefficient, 0.2–0.8 for the equilibrium allele frequency, and 0–5 for the age of the allele in coalescent units. Then we simulated population genetic variation data under the structured coalescent model conditional on the simulated trajectories^73^, using the coalescent simulator *msms*^74^. These simulations assume no recombination between the derived and the ancestral haplotype, supported by the phylogenetically conserved low rate of recombination in this region. We assumed an unscaled mutation rate^75^ of 2.2 × 10^-9^ and a generation time of 5 years^76^.

We estimate the age of the derived haplotype using an approximate Bayesian computation (ABC) approach. We computed three summary statistics from the simulated data: (1) nucleotide diversity within the ancestral haplotype, (2) nucleotide diversity within the derived haplotype, and (3) average divergence between the ancestral and the derived haplotype. To compute the same statistics from the data, we selected individuals homozygous for the small haplotype A (S1, S2, S3, S4, S5, S6, S9, S10, S11, S12) or homozygous for the large haplotype I (L1, L3, L5, L6, L12). These individuals do not have any recombinant haplotypes in the 300 Kb candidate region (TGUA1:55083662-55369631; see Fig. 2d).

### Testing for a chromosomal inversion

Chromosomal inversions are known to influence local recombination rates and thus the extent of LD due to inviable recombinant products produced in heterozygous individuals^33,77^. The sequential evolution of these strategies invokes a rare recombination event as well as drift within the inverted region. Thus, to infer the positions of breakpoints of a putative inversion, we examined the candidate and flanking regions for a deficiency in shared polymorphisms. To estimate the density of shared polymorphism and identify breakpoints, we analyzed the same small-billed and large-billed individuals that carried non-recombinant haplotypes across the candidate region (Fig. 2d). Then we calculated the density of these shared polymorphisms using kernel density estimation implemented in R (R Core Team, 2017) with a rectangular kernel and a bandwidth of 1.5 Kb (Supplementary Fig. 8). Paired-end read data allowed us to further pinpoint the location of a potential breakpoint, as read pairs that were sequenced across a breakpoint in an individual with a homozygous inversion are expected to map distantly and in the wrong orientation. Therefore, we searched for such read pairs in a homozygous, large-billed individual (L1), but did not find any signal of such an orientation. However, we found one cluster of single mapped reads to 55,075,193 bp on TGU1A, within the candidate region. We used this position as a potential left breakpoint for further PCR validation of an inversion.

To test whether the large-billed allele is inverted relative to the small-billed allele, we designed two primer sets: one spanning the left breakpoint (LBP forward: TGG ACT GGT GAT GTG AGG AG; LBP reverse: AAT GAG ACA TGG GAG GAG GA), and a second that is fully contained within the putative inversion to serve as an internal control (Internal forward: CGA GAA AGG AAC GCT TTT TG; Internal reverse: GGT TGC AGA GCA GGA AAG AC). The primers were designed based on the zebra finch reference genome, using the LBP coordinates predicted in the read-based analysis of genetic variation described above. If the large-billed allele is inverted, the LBP primers should amplify only the small-billed allele, while the internal control will amplify in all samples. The LBP and Internal primers were amplified with 1.5 µl of DNA (average 82 ng) with 5 µl of Multiplex PCR Kit master mix (Qiagen), 1 µl of each of the primer pairs at 2 µM, and 2.5 µl of molecular grade water. Cycling conditions for the LBP primers were: 95 °C for 15 min, 45 cycles of (94 °C for 30 s, 57 °C for 90 s, 72 °C for 90 s), 72 °C for 10 min, and hold at 4 °C. Cycling conditions for the Internal primers were: 95 °C for 15 min, 40 cycles of (94 °C for 30 s, 57 °C for 90 s, 72 °C for 60 s), 60 °C for 30 min, and hold at 4 °C. All PCR products were visualized on a 1% agarose gel with a Routine 100 bp low scale DNA ladder (Fisher Scientific). For the LBP primer pair, in silico PCR based on the zebra finch reference genome predicts an amplicon size of 201 bp in small-billed individuals only, while the Internal primer pair should yield an amplicon size of 463 bp for all individuals.

To compare the sequences of the small- and large-bill alleles, we generated a draft assembly of the non-wildtype allele carried by large-billed individuals. We sequenced a sample of high molecular weight genomic DNA from a single large-billed individual (L5), previously identified in the *LDna* analysis as homozygous for the large-billed allele, on six PacBio SMRT cells at the UC Davis DNA Technologies Core facility. We filtered these reads by aligning to the zebra finch (taeGut 3.2.4) using *BWA-MEM*^78^ with default settings, and only retained reads that mapped to the candidate region (TGU1A: 54.9–55.5 Mb). We then assembled the single-end and paired-end Illumina reads from the targeted resequencing effort for the same individual into contigs using *SPAdes*^79^. All contigs larger than 1 Kb were scaffolded using the filtered PacBio reads with *SSPACE-LongRead*^80^. For comparison, we constructed additional assemblies using three alternative algorithms: (1) *BLASR*^81^ for read mapping to the zebra finch reference; (2) performing a hybrid assembly using *SPAdes* with both Illumina and PacBio reads; and (3) using *CAP3*^82^ to perform a meta-assembly with the assembled contigs. To compare the assembled large-billed allele with the small-billed allele and assess for inversions, we mapped all large-billed scaffolds > 5 Kb to the candidate region in the zebra finch reference and generated a dot plot in *Gepard*^83^.

### Comparative analysis of allelic variation with Darwin’s finches

To determine if the genes in the candidate region of *P. ostrinus* are also commonly found to influence bill size and shape in other bird species, we conducted a comparative analysis of a region homologous in Darwin’s finches (*Geospiza*) genome. If the candidate region of high LD and the genes contained within have a conserved function in shaping bill morphology, we expect that to find that previous genome-wide scans would have identified this region and subset of same genes. With the possibility that this chromosomal region and *IGF1* have remained undetected in previous studies, and if genetic variation in this candidate region is preserved independent of phylogeny, we expect genetic similarities within the candidate region would resolve bill morphology and not species histories. To determine if any alleles associated with bill morphology in *P. ostrinus* are also segregating or fixed within the highly-studied *Geospiza* species, we obtained publically available paired-end (2 × 100 nt) whole-genome sequence data for four individuals from *G. fuliginosa* (small ground finch), two *G. fortis* (medium ground finch), and four *G. magnirostris* (large ground finch)^25^ (Supplementary Table 11). Reads from these three Darwin’s finch species and this study’s targeted resequence data from small- and large-billed *P. ostrinus* morphs were mapped to the *G. fortis* genome using *stampy*. We followed the aforementioned methods to identify SNP variants with *ANGSD* to identify SNP variants across both genera, and assessed allele sharing and variation across the candidate region and conducted a PCA using *flashPCA* on the collectively called SNP genotypes.

### Population genetic analysis of the three bill morphs

We collected additional targeted resequencing data on 21 mega-billed adult birds (LMW = 19 m6 ± 0.9 mm) for the candidate region on TGU1A. We collected and processed sequence data following methods detailed above, combining all bill morphs for subsequent analyses. We used *ANGSD* to call SNP genotypes from the BAM files with a minimum depth of 30-fold sequence coverage, a minimum mapping quality 20, and a minimum variant quality of 60. SNPs where filtered to retain sites with a maximum of 10% missing data and a minimum minor allele frequency of 3%. All downstream analysis contained only the SNPs and sequence that was mapped within the coordinates of our target region. We estimated observed heterozygosity (*H*_O_) for each phenotype using *PLINK* v1.9 and conducted a PCA using *flashPCA* on SNP genotypes. To assess haplotype patterns within this targeted 500 Kb region of interest, we phased SNP genotypes using *SHAPEIT* following methods described in the main text (i.e., using a reduced recombination rate^27^). We constructed and viewed unrooted  neighbor-joining (NJ) haplotype networks in *SplitsTree*.

### Reporting summary

Further information on research design is available in the Nature Research Reporting Summary linked to this article.

## Electronic supplementary material

Supplementary Information

Description of Additional Supplementary Files

Supplementary Data 1

Supplementary Data 2

Reporting Summary

## Data Availability

Demultiplexed and processed (i.e., trimmed and clipped) FASTQ files are available on NCBI SRA (accession SRP140635 [https://www.ncbi.nlm.nih.gov/sra/SRP140635]) for the three pools used in Pool-seq (small-, large-, and mega-billed morphs) and for the targeted capture resequencing of 12 small- and 12 large-billed individuals. We further deposited their respective BAM files, mapped to the reference taeGut2 genome.
