# Supplementary material for: Growth factor gene IGF1 is associated with bill size in the black-bellied seedcracker Pyrenestes ostrinus
Source: Nat Commun. 2018 Nov 19;9:4855. doi: 10.1038/s41467-018-07374-9 (PMC6242981; doi:10.1038/s41467-018-07374-9)
Supplement: Supplementary file 1 — Supplementary Information [file 41467_2018_7374_MOESM1_ESM.pdf]

Supplementary Information

Growth factor gene IGF1 is associated with bill size in the black-bellied seedcracker (*Pyrenestes ostrinus*)

vonHoldt et al.

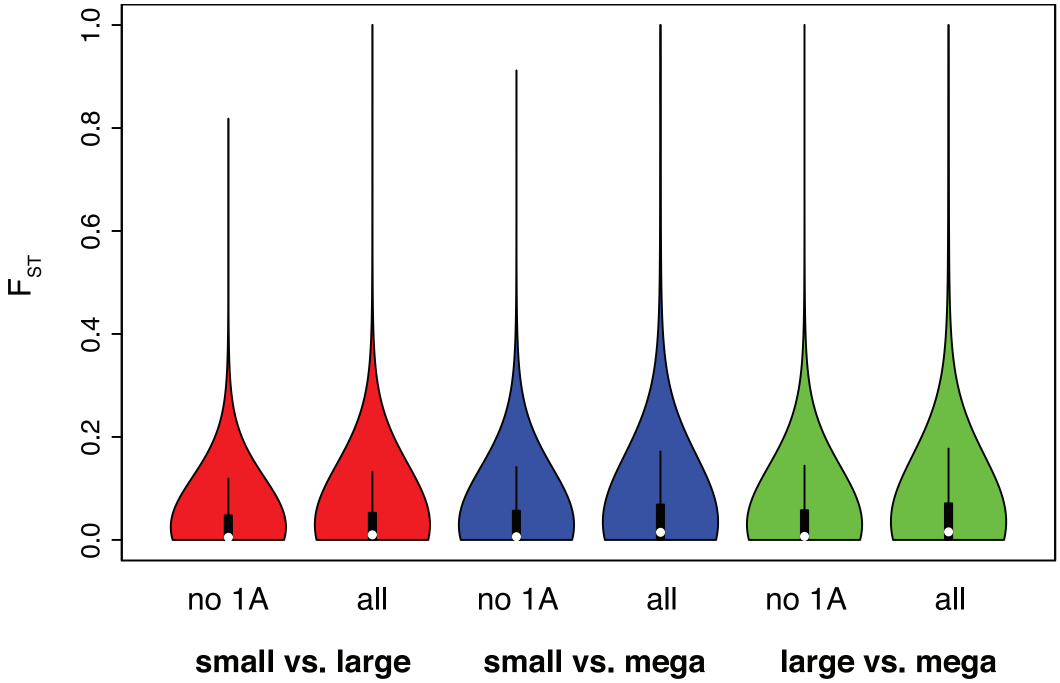

Supplementary Figure 1. Distribution of pairwise F_{ST} values for all chromosomes except TGU1A and all chromosomes (labeled as "no 1A" and "all", respectively), calculated based on a sliding window approach (size=200bp, step=200bp) from pooled sequencing for the three bill morphs.

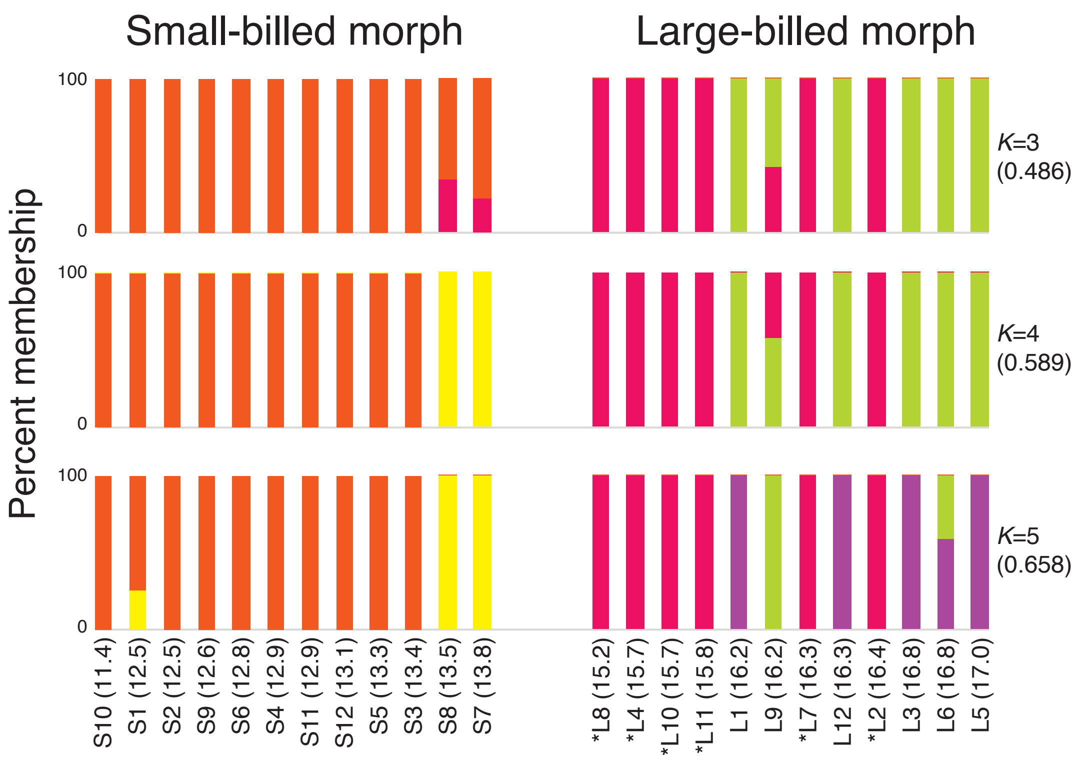

Supplementary Figure 2. Population structure analysis (*K*=3-5) using 4,717 SNPs from TGU1A. Sample IDs (LMW in parentheses) are along the X-axis, with birds ordered from smallest LMW (left) to largest LMW (right, in millimeters). The cross-validation value is indicated in parentheses next to each *K* partition value. Asterisks indicate that large-billed individuals are members of the heterozygous subgroup.

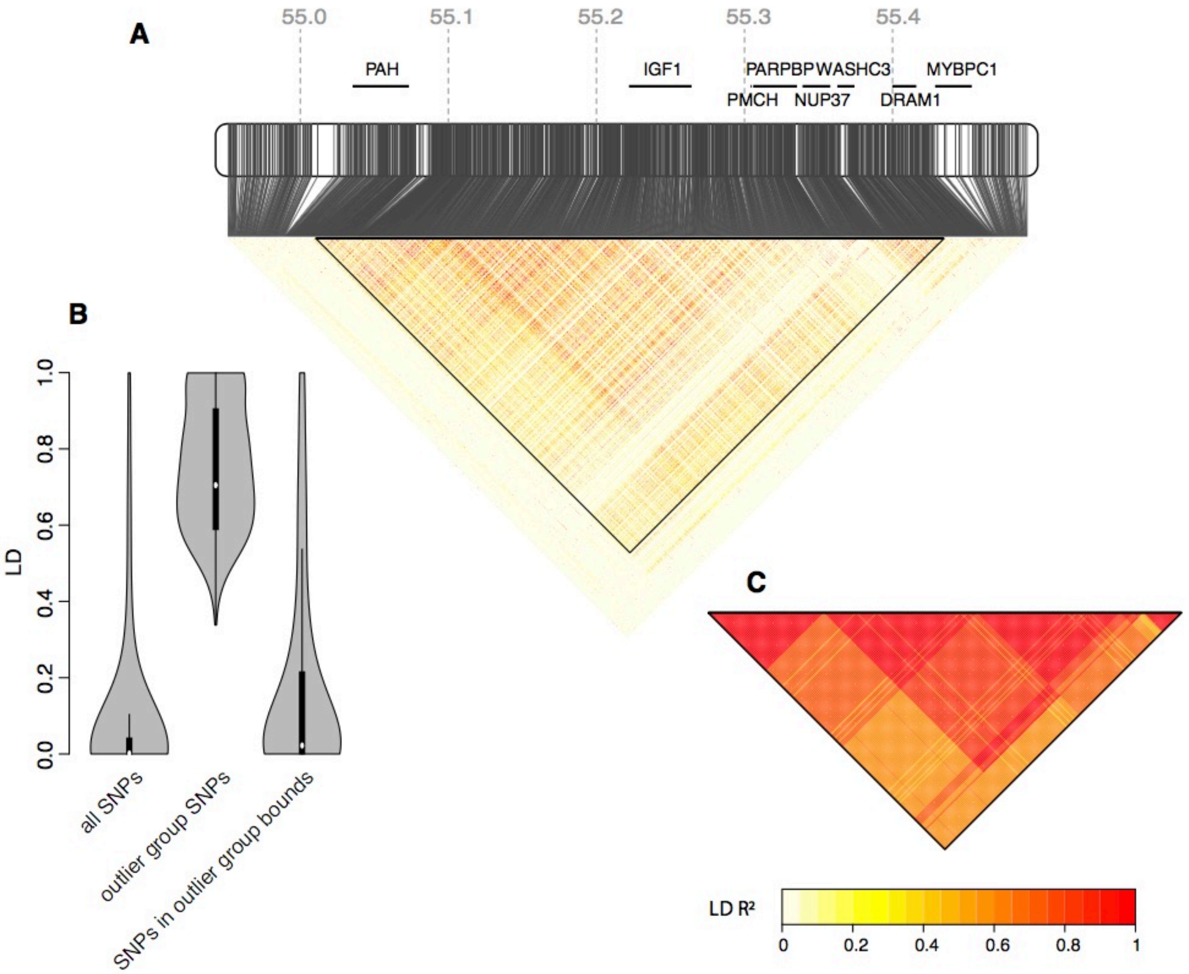

Supplementary Figure 3. Linkage disequilibrium (LD) in small- and large-billed *P. ostrinus* morphs on zebra finch chromosome 1A. **A)** LD heat map of all SNPs in the target and candidate region associated with bill morphology. The black triangle marks the bounds of the highly linked "outlier group" SNPs. **B)** Violin plots of LD for: all SNPs; all highly-linked "outlier group" SNPs; and all SNPs within the bounds of the "outlier group" SNPs (black triangle in panel A). **C)** LD heat map of "outlier group" SNPs only.

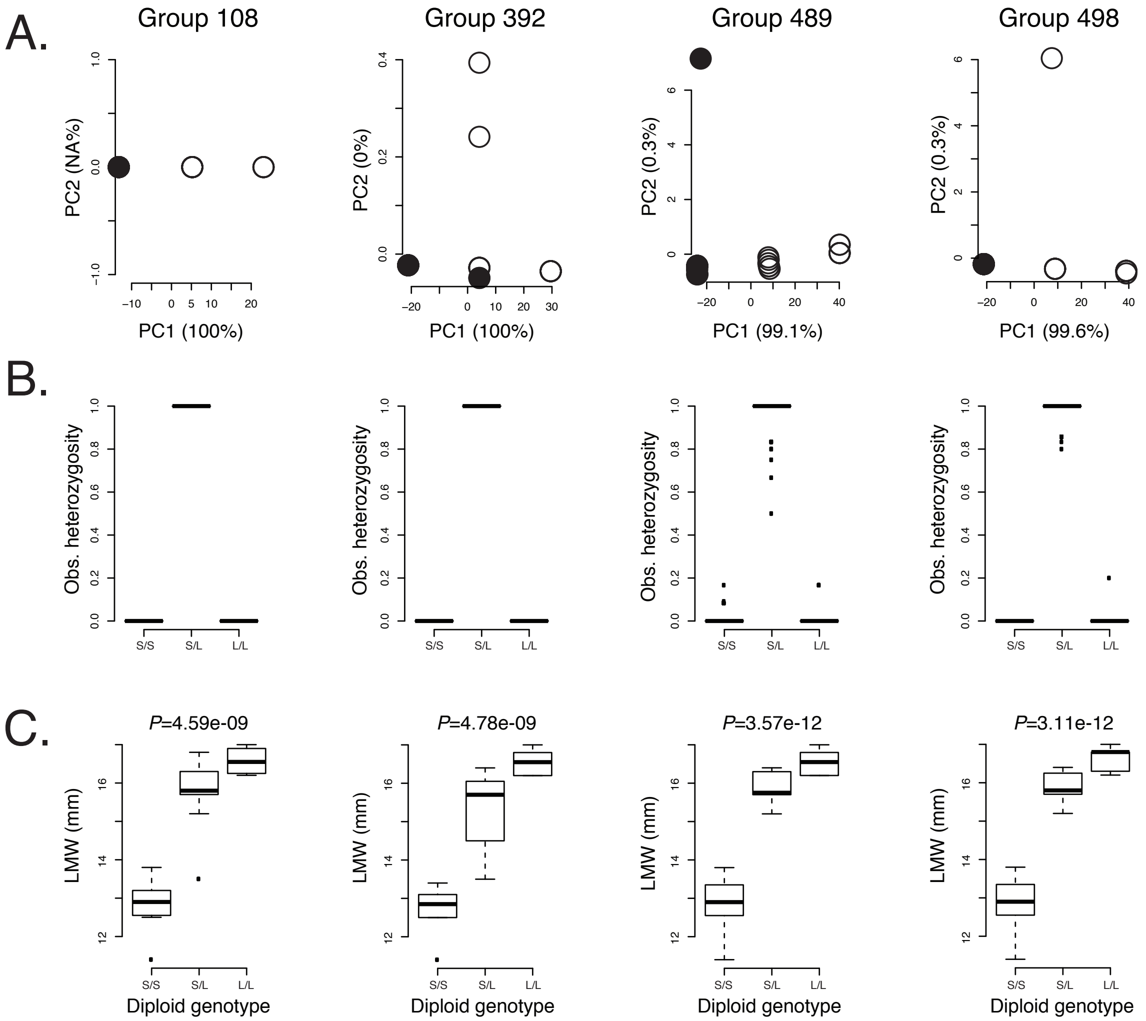

Supplementary Figure 4. Genetic diversity and phenotypic associations for each of the four groups of loci with high linkage disequilibrium (LD group 108, 392, 489, and 498) using **A)** PCA of genotypes in each LD group (percent variance explained in parentheses; filled circles represent small-beaked morphs; open circles points represent large-beaked morphs), note that there is no PC2 variance for group 108, **B)** observed heterozygosity for each multi-locus genotype identified in the PCA (allele S is associated with the small-bill morphology; allele L is associated with the large-bill morphology), and **C)** diploid genotypes with respect to lower mandible width (LMW) and ANOVA *P*-values are provided. Small beaked individuals all have LMW<14mm.

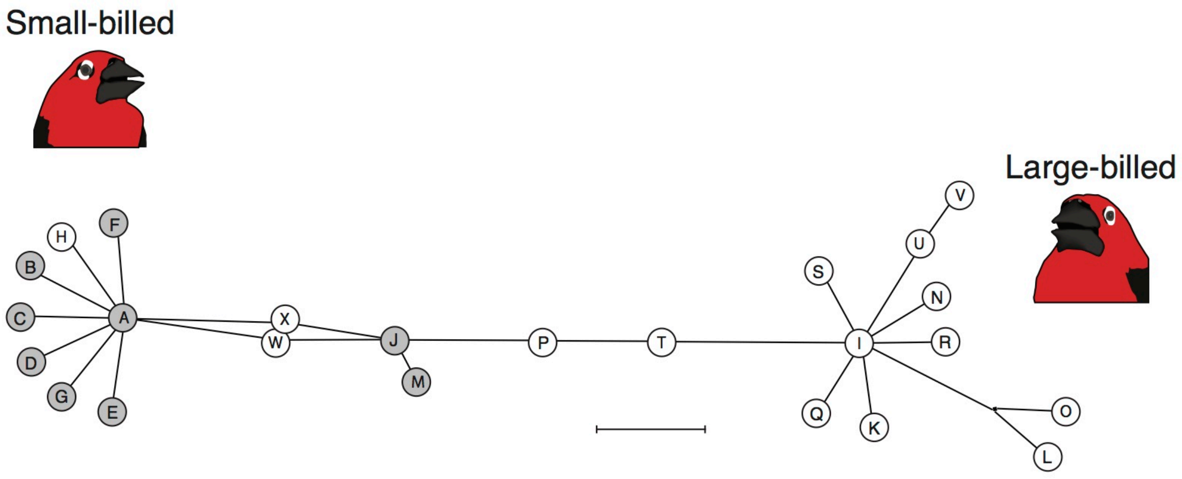

Supplementary Figure 5. Median joining haplotype tree for loci with high linkage disequilibrium. Shaded symbols indicate haplotypes associated with small-bill size. A genetic distance scale indicates a unit of 100. Haplotype frequencies are also provided for each bill morphology in Supplementary Table 8.

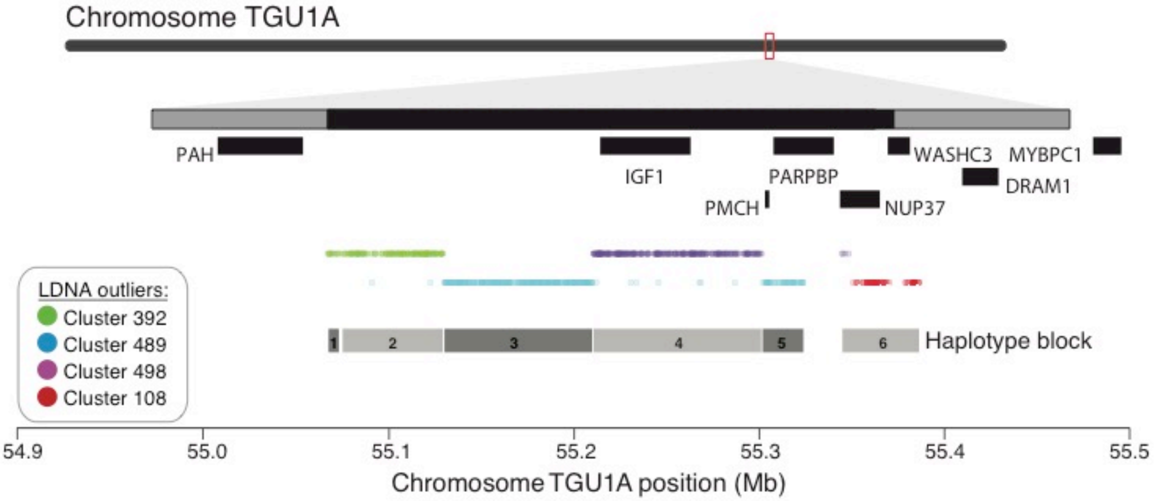

Supplementary Figure 6. *LDna* outlier clusters in the candidate region align with haplotype blocks. Density plots of SNPs within each *LDna* outlier cluster (green, blue, purple, and red points) identified through network analysis of linkage disequilibrium, and haplotype blocks (numbered, dark and light gray segments) identified by analysis of phased haplotypes. The schematic of the candidate and flanking regions and annotated genes are as in Fig. 2A.

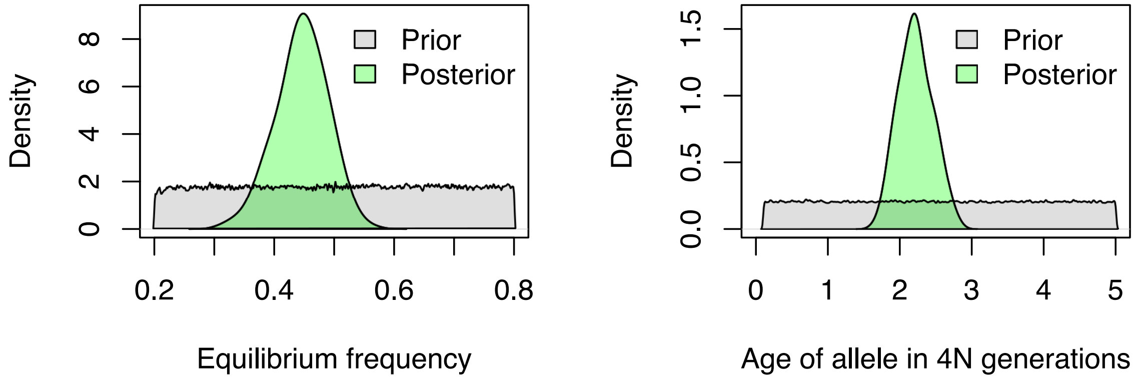

Supplementary Figure 7. *Approximate Bayesian Computation* estimation of equilibrium frequency (left panel) and age (right panel) of the non-recombinant (NR) haplotype I associated with bill size.

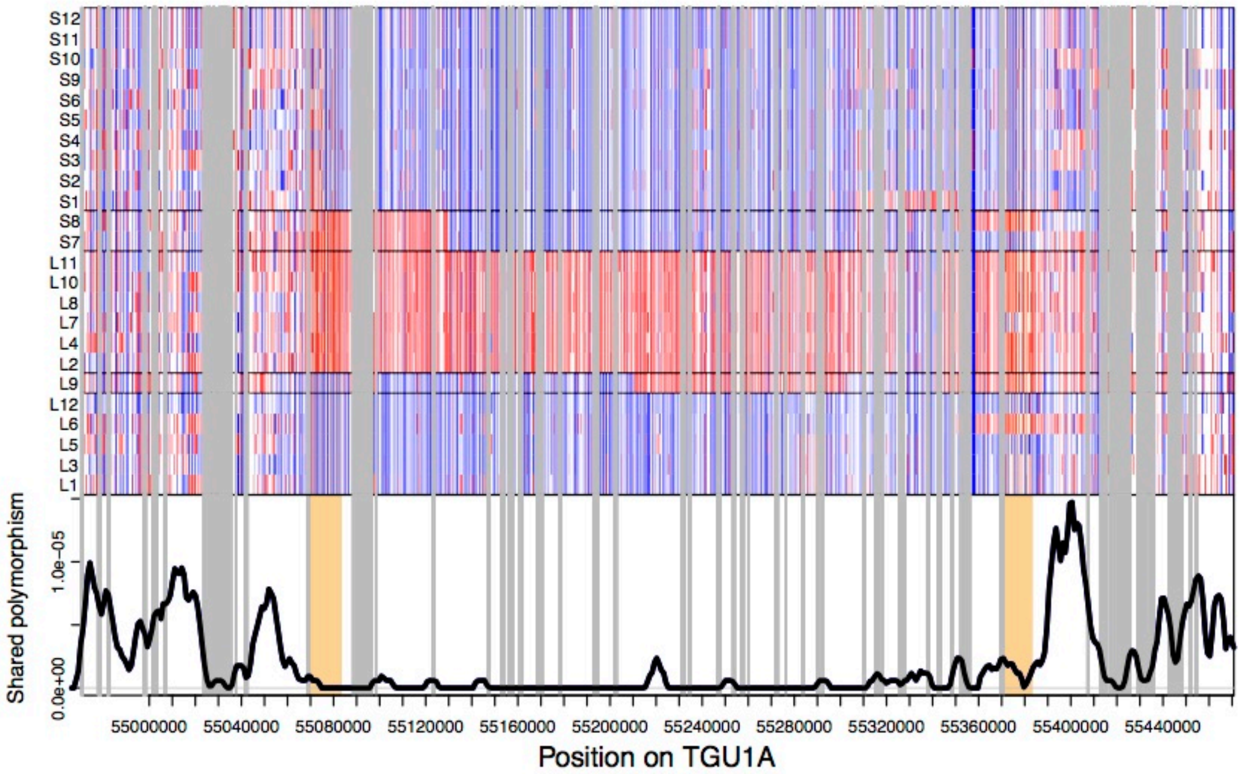

Supplementary Figure 8. Identifying the location of two potential inversion breakpoints in the candidate region, based on shared polymorphisms. Genotypes are plotted along the genomic position, with the homozygous reference genotype plotted in white, heterozygous genotype in red, and homozygous non-reference genotype in blue. The density of shared polymorphisms between small-billed birds (S1, S2, S3, S4, S5, S6, S9, S10, S11, S12) and large-billed birds (L1, L3, L5, L6, L12) is plotted at the bottom and shows a 300kb large region depleted of shared polymorphisms. Grey bars indicate low mapping quality (coverage<50). The two orange bars show confidence intervals of two potential breakpoints, predicted based on linkage disequilibrium using the software *LDna*.

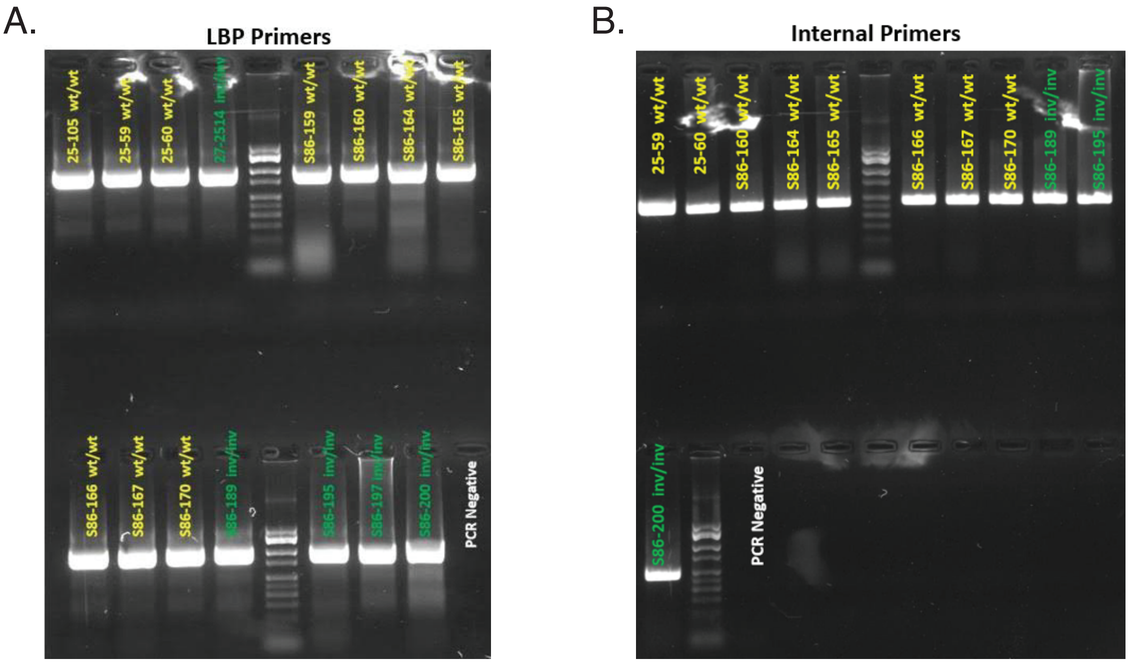

Supplementary Figure 9. Gel images for PCR validation of a potential inversion in large-billed birds. **A)** Left break point (LBP; product size 750-1000bp) and **B)** internal primers (product size ~ 450bp), with a Routine 100bp low scale DNA ladder (Fisher Scientific) in the middle column of each gel. Ladder bands are, in increasing order: 50bp, 100bp, 200bp, 300bp, 400bp, 500bp, 750bp, 1000bp, 1400bp, 1550bp, and 2000bp. Sample IDs in yellow are small-billed individuals, while green indicates large-billed individuals. LBP product sizes are consistent with *in silico* PCR based on the draft assembly of the large-bill allele, which predicts a 950bp PCR product.

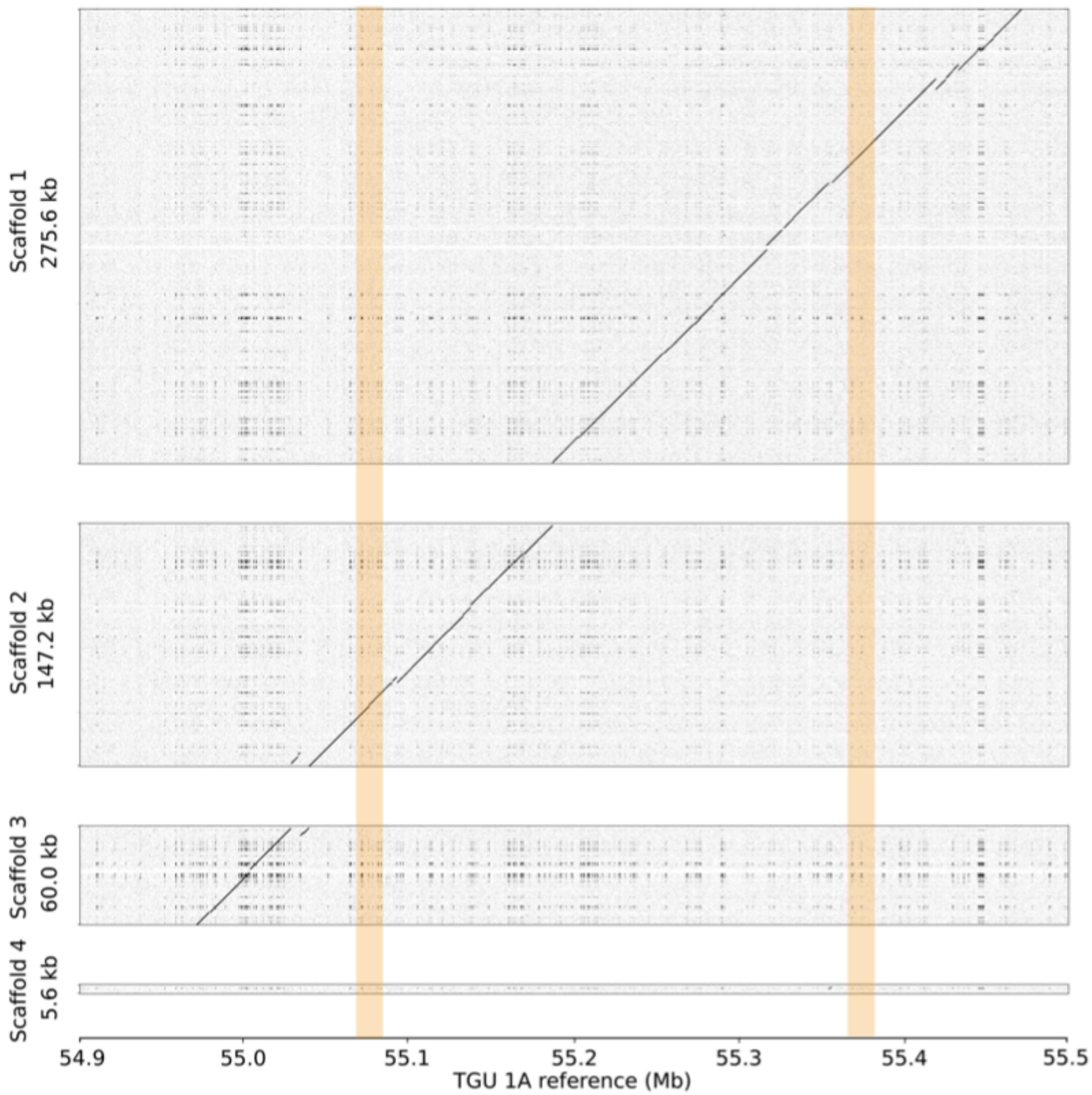

Supplementary Figure 10. Dot plot of the four largest scaffolds that map to the candidate region in the zebra finch genome (TGU1A, 54.9-55.5Mb). Predicted breakpoint positions from the software *LDna* are shown as orange bars (see Supplementary Fig. 8). Note that scaffold 2 covers the left putative breakpoint, and scaffold 1 covers the right putative breakpoint. An inversion would lead to a discontinuity in the mapping of the scaffold to the reference sequence at the breakpoint positions, thus the dot plot does not support the presence of an inversion.

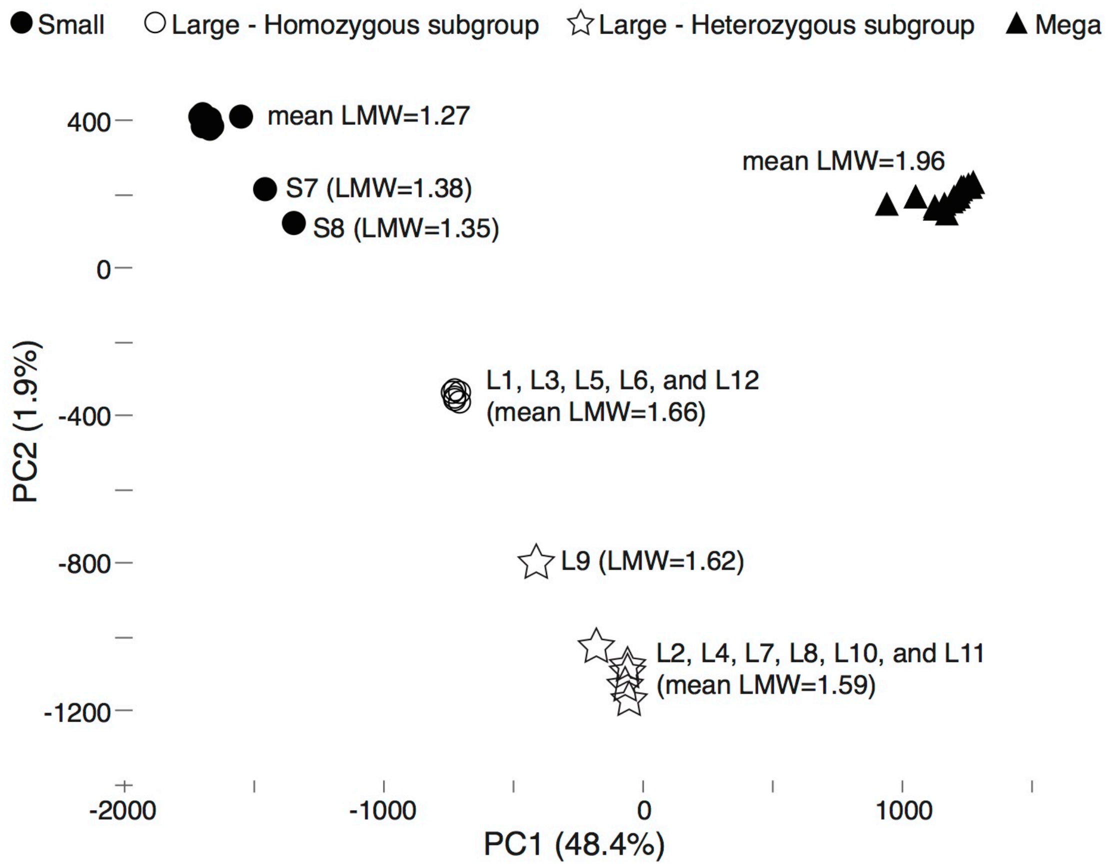

Supplementary Figure 11. A principal component analysis of genetic variation across 5,411 SNP genotypes from the target region (TGU1A: 54.9-55.5Mb). The lower mandible width (LMW in mm) is provided for each cluster, with singletons labeled with sample ID and LMW (See Supplementary Data 2 for more details). The proportion of variance explained by each component is provided along each axis. The genotypic subgroups of the large-billed individuals are indicated.

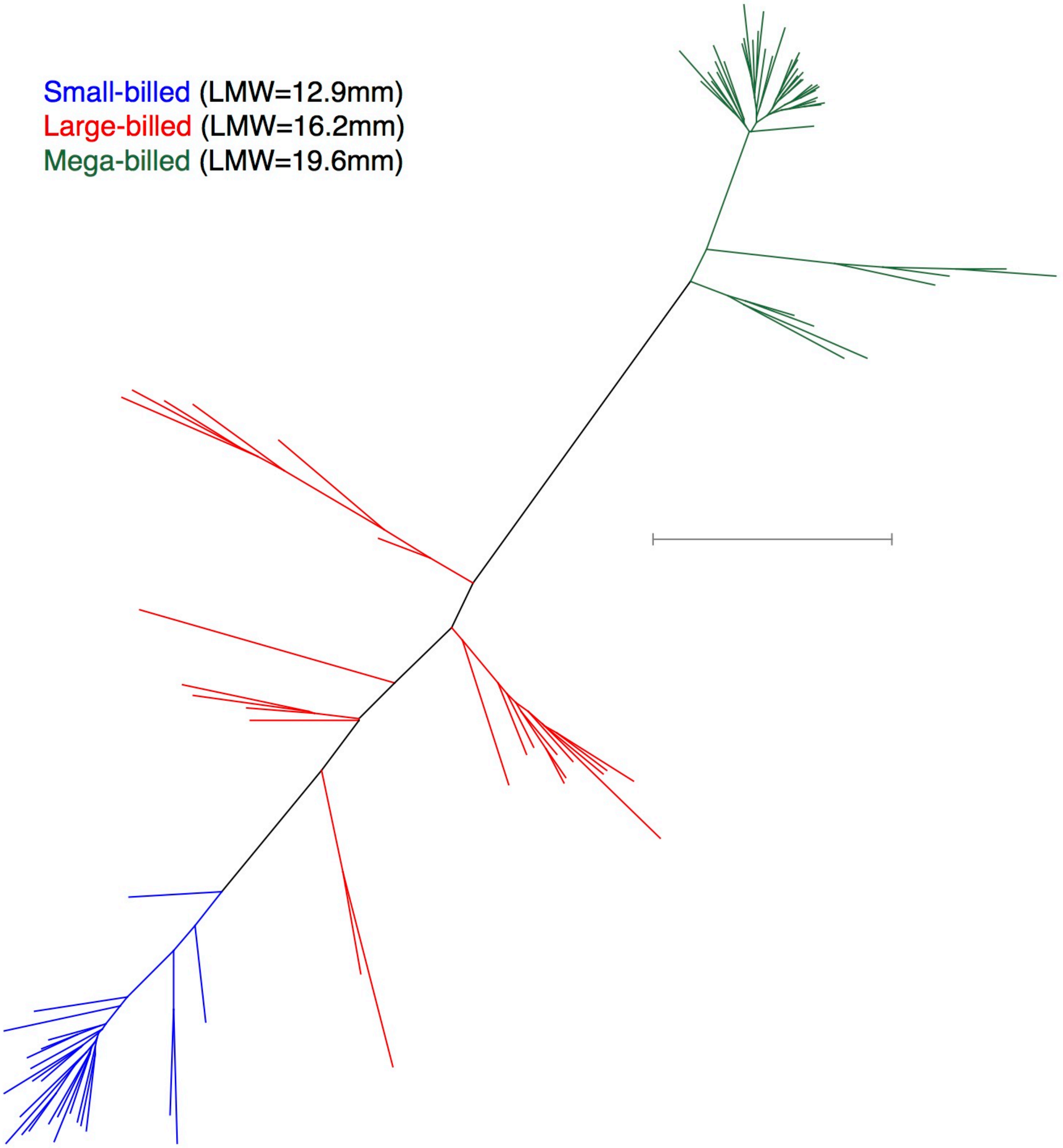

Supplementary Figure 12. A neighbor joining tree constructed from phased genotypes across 5,411 SNPs and three *P. ostrinus* morphs (colors are indicated in figure). Average lower mandible width (LMW) are provided for each bill morph in parentheses. Genetic scale bar of 0.1 units is provided.

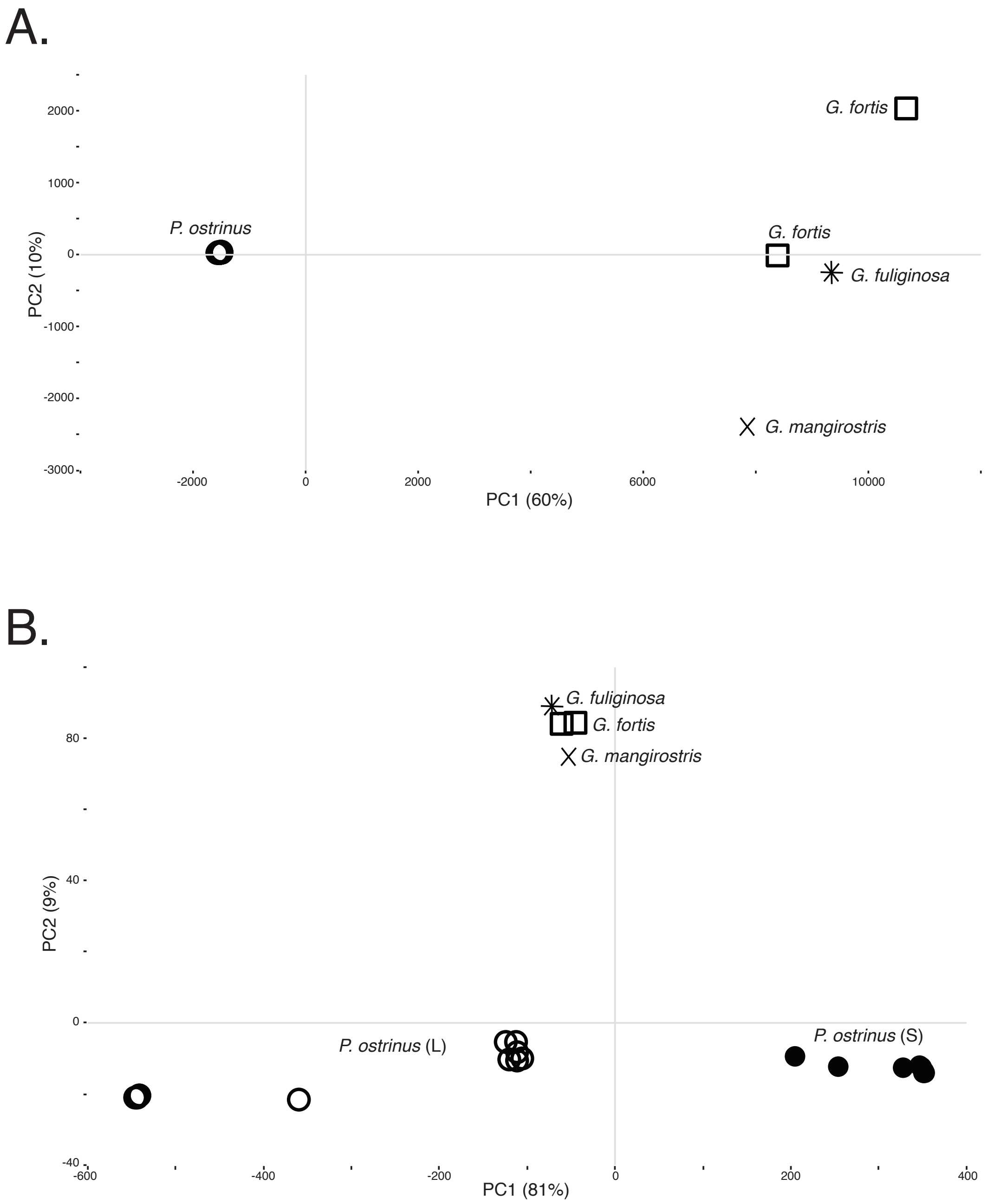

Supplementary Figure 13. Principal component analysis of **A)** of 9,991 sites mapped to scaffold 10 of *G. fortis* and **B)** 755 highly divergent sites (F_{ST}>0.5) between small- (S) and large- (L) billed *P. ostrinus*, also mapped to scaffold 10 of *G. fortis*.

Supplementary Table 1. The number of 200bp windows with F_{ST}>0.8 in pairwise comparisons among morphs. (Abbreviations: S, small-billed; L, large-billed; M, mega-billed)

TGU chr	S vs. L	S vs. M	L vs M
1	0	1	1
1A	13	1464	1088
1B	0	0	0
2	0	3	2
3	1	4	3
4	0	0	1
4A	0	2	0
5	0	2	1
6	0	0	0
7	0	0	0
8	0	0	2
9	0	2	3
10	0	0	0
11	0	0	0
12	0	0	0
13	0	0	1
14	0	1	0
15	0	0	0
17	0	0	1
18	0	0	0
19	0	0	0
20	0	0	0
21	0	1	0
22	0	0	0
23	0	0	0
24	0	0	0
25	0	0	0
26	0	0	0
27	0	0	0
28	0	0	0
LG2	0	0	0
LGE22	0	0	0
Z	0	3	5

Supplementary Table 2. Divergence between mega and non-mega morphs. Distribution across all zebra finch chromosomes of SNPs fixed for alternate alleles between small and mega morphs (S vs. M fixed diffs) and between large and mega morphs (L vs. M fixed diffs). (Abbreviations: chr, chromosome; S, small-billed; L, large-billed; M, mega-billed)

TGU chr	S vs. M fixed diffs	L vs. M fixed diffs	S vs. L fixed diffs
1	1	3	0
1A	2270	1647	8
1B	0	0	0
2	1	2	0
3	1	0	0
4	0	0	0
4A	0	0	0
5	0	0	0
6	0	0	1
7	0	0	0
8	0	0	0
9	0	0	0
10	0	0	0
11	0	1	0
12	0	1	0
13	0	0	0
14	0	0	0
15	0	0	0
17	0	0	0
18	0	0	0
19	0	0	0
20	0	0	0
21	0	0	0
22	0	0	0
23	0	0	0
24	0	0	0
25	0	0	0
26	0	0	0
27	0	0	0
28	0	0	0
LG2	0	0	0
LGE22	0	0	0
Z	1	1	0

Supplementary Table 3. Distribution and effect of SNPs with fixed differences between mega and non-mega (small- and large-billed) *P. ostrinus* morphs within the candidate region on TGU1A. Total number of fixed differences within each gene (SNPs); length of each gene; number of SNPs with moderate phenotypic impact ($n_{moderate}$), which includes missense variants; number of SNPs with a modifying impact ($n_{modifier}$), which includes downstream, upstream, and intron variants; and number of SNPs with low impact (n_{low}), which includes synonymous and intron splice variants. Genes in bold are significantly enriched ($P < 0.05$) in SNPs based on 10,000 bootstrap replicates.

Gene name	SNPs	Gene length (bp)	$n_{Moderate}$*	$n_{Modifier}$**	n_{Low}***
ADCK2	3	8364	6	6	0
BCAT1	1	36,977	0	1	0
BRAF	5	53,220	0	6	0
CCDC77	1	10,090	1	2	0
CCDC91	238	109,026	2	261	0
CHPT1	1	11,778	0	1	0
DDX11	25	16,214	0	34	3
DENND2A	1	32,714	0	1	0
DRAM1	2	18,212	0	3	0
HIPK2	3	86,938	0	3	0
IGF1	33	47,139	0	37	0
IPO8	4	47,190	0	8	0
IQSEC3	7	33,239	0	13	0
KDM5A	1	47,635	0	1	0
KDM7A	1	32,487	0	3	0
KLHL42	11	11,515	0	15	0
MANSC4	0		0	6	0
MRPS33	0		0	2	0
MYBPC1	2	118,788	0	4	0
NUP37	11	21,091	0	15	0
PAH	1	42,296	0	2	0
PARPBP	11	33,856	0	11	1
PMCH	0		0	2	0
PTHLH	5	5,910	0	9	0
RECQL	1	11,623	0	1	0
SOX5	1	242,393	0	1	0
WASHC3	18	13,314	0	29	0
WNK1	5	97,065	0	4	1

*Includes: Missense variants; **Includes: Downstream, intron, and upstream variants; ***Includes: Synonymous and intron splice region variants

Supplementary Table 4. Genes located within the targeted region on chromosome TGU1A, the number of SNPs within their boundaries, and the corresponding median *P*-value from the quantitative association analysis of the small- and large-billed individuals (abbreviations S and L, respectively). Bolded genes are considered candidates for influencing beak morphology (*P*<0.05).

Ensembl Gene name	SNP count	Median *P*-value for S/L
PAH	161	0.227
IGF1	468	6.45×10^{-6}
PMCH	16	0.055
PARPBP	374	0.108
NUP37	202	1.46×10^{-6}
WASHC3	130	1.58×10^{-4}
DRAM1	38	0.314
MYPBC1	127	0.355

Supplementary Table 5. Groups of loci with high linkage disequilibrium (LD) identified in small- and large-billed *P. ostrinus*. (Abbreviations: median absolute deviation, MAD; number of loci, nLoci; number of edges, nE)

Outlier cluster name	Merge at	nLoci	nE	λ	Median LD	Unscaled MAD LD
82	0.85	172	14706	48.90	0.999	0
108*	0.83	90	4005	36.92	0.999	0
352	0.42	42	861	41.64	0.992	0
392*	0.86	214	22581	62.93	0.999	0
412	0.9	55	1485	54.31	0.988	0
455	0.26	37	666	36.52	0.988	0
457	0.26	126	7875	124.42	0.988	0
478	0.28	92	4099	90.84	0.988	0
480	0.28	42	717	41.47	0.988	0
481	0.28	46	992	45.42	0.988	0
484	0.28	43	862	42.45	0.988	0
489*	0.87	373	53582	61.33	0.999	0
490	0.87	83	2122	8.50	0.999	0
498*	0.87	295	38914	48.53	0.999	0
513	0.85	73	2278	35.72	0.999	0
527	0.8	142	7985	111.61	0.999	0
708	0.6	41	492	8.43	0.609	0.206

* Outlier clusters exhibit patterns consistent with a diallelic locus segregating into heterozygous and alternate homozygous genotypes: three genotypic groups (assessed by PCA), with nearly all loci heterozygous for the intermediate group (representing heterozygous individuals) and nearly all loci homozygous for the groups at the extremes (representing individuals homozygous for alternate alleles).

Supplementary Table 6. Number of pairwise differences between the haplotypes containing 972 loci with high linkage disequilibrium.

Haplotype	B	C	D	E	F	G	H	I	J	K	L	M	N	O	P	Q	R	S	T	U	V	W	X
A	2	5	4	2	2	21	8	972	215	970	969	304	965	965	582	971	971	958	882	953	949	21	21
B		7	6	4	4	23	10	970	217	968	967	306	963	963	580	969	969	956	880	951	947	23	23
C			9	7	7	26	13	967	220	965	964	309	962	960	581	966	968	953	877	950	952	26	26
D				6	6	25	12	968	219	966	965	308	691	961	587	967	967	954	878	949	945	25	25
E					4	23	10	970	217	968	967	306	963	963	580	969	969	956	880	951	947	23	23
F						23	10	970	217	968	967	306	963	963	584	969	969	956	880	951	947	23	23
G							29	951	236	949	948	325	944	944	561	950	950	937	861	932	928	42	42
H								964	221	962	963	310	957	959	576	963	963	950	874	945	942	29	29
I									757	2	3	668	7	7	390	1	1	14	90	19	23	951	951
J										759	756	93	754	750	367	756	756	743	667	772	770	202	202
K											5	668	9	9	392	3	3	16	92	21	25	949	949
L												667	10	8	389	4	4	17	93	22	26	948	948
M													665	661	458	667	667	654	758	683	681	291	291
N														12	395	8	8	21	97	26	26	946	946
O															393	8	8	21	97	26	28	948	946
P																389	389	376	300	409	409	565	565
Q																	2	15	91	20	24	950	950
R																		15	91	20	22	950	950
S																			104	33	37	937	937
T																				109	113	861	861
U																					8	966	966
V																						962	962
W																							2

Supplementary Table 7. Frequencies and individual genotypes for the 24 haplotypes based on 972 SNPs within the candidate region in high linkage disequilibrium. Haplotypes were assigned to a haplogroups based on their sequence similarities. An asterisk * indicates that a large-billed morph was both heterozygous for the haplogroups and belonged to the heterozygous subgroup of large-billed individuals. (Abbreviations: large-billed, L; n, count of chromosomes; small-billed, S)

Haplotype	Haplogroup	n_S	n_L	*f*(S)	*f*(L)	Homozygous in individuals:	Heterozygous in individuals:
A	S	18	1	0.75	0.04	S2-4, S10-12	S1, S5-9, L7*
B	S	1	0	0.04	0.00		S6
C	S	0	1	0.00	0.04		L4*
D	S	1	0	0.04	0.00		S9
E	S	1	0	0.04	0.00		S5
F	S	0	1	0.00	0.04		L8*
G	S	1	0	0.04	0.00		S1
H	S	0	1	0.00	0.04		L11*
I	L	0	6	0.00	0.25	L3, L5	L1, L6
J	S	1	0	0.04	0.00		S7
K	L	0	2	0.00	0.08	L12	
L	L	0	1	0.00	0.04		L11*
M	S	1	0	0.04	0.00		S8
N	L	0	1	0.00	0.04		L8*
O	L	0	1	0.00	0.04		L4*
P	L	0	1	0.00	0.04		L9
Q	L	0	1	0.00	0.04		L1
R	L	0	1	0.00	0.04		L7*
S	L	0	1	0.00	0.04		L9
T	L	0	1	0.00	0.04		L6
U	L	0	1	0.00	0.04		L10*
V	L	0	1	0.00	0.04		L2*
W	S	0	1	0.00	0.04		L10*
X	S	0	1	0.00	0.04		L2*

Supplementary Table 8. Genomic coordinates on TGU1A for the six recombinant regions and their respective quantitative associations with LMW. (Abbreviation: recomb., recombination)

Recomb. block	Start	Stop	Regression coefficient (ß)	Regression R^2	Wald's Test (T)	Average -log(*P*-value)	Genes
1	55067422	55073047	0.197±0.02	0.79	9.0	7.903	
2	55075069	55129215	0.195±0.02	0.83	10.2	7.903	
3	55130138	55209945	0.206±0.02	0.83	10.0	8.841	
4	55210684	55301494	0.195±0.02	0.83	10.2	8.682	*IGF1*
5	55302167	55324218	0.206±0.02	0.83	10.0	8.841	*PMCH*, *PARPBP*
6	55345289	55386717	0.204±0.02	0.79	8.9	7.861	*NUP37*, *WASHC3*

Supplementary Table 9. Predicted phenotypic effects of each SNP in the region of 972 lo[illegible] with high linkage disequilibrium (LD). Gene size was estimated as the coordinates of the fi[illegible] and last SNP annotated within the gene's boundaries by *Ensembl's VEP*.

LD block	Annotation	Gene	Gene size (bp)	Number of SNPs	Impact
1	Intron	*PAH*	15,975	17	Modifier
2	Intergenic	--		148	Modifier
2	Downstream	*PAH*	15,975	18	Modifier
2	Intron	*PAH*	15,975	28	Modifier
2	Synonymous	*PAH*	15,975	1	Modifier
2	Missense	*PAH*	15,975	1	Modifier
3	Intergenic	--		309	Modifier
4	Upstream	*IGF1*	46,587	33	Modifier
4	Intron	*IGF1*	46,587	146	Modifier
4	Missense	*IGF1*	46,587	1	Moderate
4	3' UTR	*IGF1*	46,587	1	Modifier
4	Downstream	*IGF1*	46,587	23	Modifier
4	Intergenic	--		91	Modifier
4	Upstream	*PMCH*	683	2	Modifier
5	Upstream	*PMCH*	683	12	Modifier
5	Missense	*PMCH*	683	3	Moderate
5	Downstream	*PMCH*	683	6	Modifier
5	Intron	*PARPBP*	15,800	31	Modifier
5	Missense	*PARPBP*	15,800	1	Moderate
6	Upstream	*NUP37*	20,061	2	Modifier
6	Intron	*NUP37*	20,061	50	Modifier
6	Intron	*WASHC3*	12,753	8	Modifier
6	Splice region	*NUP37*	20,061	1	Low
6	Missense	*NUP37*	20,061	1	Moderate
6	Synonymous	*NUP37*	20,061	2	Low
6	Downstream	*NUP37*	20,061	11	Modifier
6	Missense	*WASHC3*	12,753	1	Moderate
6	Downstream	*WASHC3*	12,753	18	Modifier

Supplementary Table 10. Results of bootstrap test for enrichment of SNPs predicted to affect genes in the candidate region: Predicted proportion of SNPs assuming equal distribution among genes while accounting for gene size; actual proportion of SNPs observed to be associated with each gene; and *P*-value based on 10,000 resampling iterations. Gene size is estimated based on Supplementary Table 9. (Abbreviations: No., number; prop., proportion)

Gene name	Gene size (bp)	No. of SNPs	Predicted prop. of SNPs	Actual prop. of SNPs	*P*-value
IGF1	46,587	204	0.42	0.49	0.001
NUP37	20,061	67	0.18	0.16	0.830
PAH	15,975	65	0.14	0.16	0.206
PARPBP	15,800	32	0.14	0.08	1.000
PMCH	683	23	0.01	0.01	0.000
WASHC3	12,753	27	0.11	0.06	0.999

Supplementary Table 11. Data for pair-end (2x100nt) genome sequence data from *Geospiza* analyzed in this study and downloaded from EMBL-EBI (www.ebi.ac.uk).

SRR identifiers	Experiment SRX identifiers	Sample ID	Species	Bill morphology
1607462, 1607463	728505, 728506	ful1	*fuliginosa*	Small
1607464, 1607465	728507, 728508	ful2	*fuliginosa*	Small
1607466, 1607467	728509, 728510	fulig12	*fuliginosa*	Small
1607468, 1607469	728511, 728512	fulig15	*fuliginosa*	Small
1607458, 1607459	728501, 728502	F1	*fortis*	Medium
1607460, 1607461	728503, 728504	F2	*fortis*	Medium
1607486, 1607487	728529, 728530	MG2	*magnirostris*	Large
1607488, 1607489	728531, 728532	MG3	*magnirostris*	Large
1607490, 1607491	728534, 728535	MG4	*magnirostris*	Large
1607492, 1607493	728535, 728536	MG7	*magnirostris*	Large
