# Supplementary material for: Growth factor gene IGF1 is associated with bill size in the black-bellied seedcracker Pyrenestes ostrinus
Source: Nat Commun. 2018 Nov 19;9:4855. doi: 10.1038/s41467-018-07374-9 (PMC6242981; doi:10.1038/s41467-018-07374-9)
Supplement: Supplementary file 2 — Description of Additional Supplementary Files [file 41467_2018_7374_MOESM2_ESM.pdf]

Description of Additional Supplementary Files

File Name: Supplementary Data 1
Description: Predicted regions of selective sweeps for each bill morph estimated from pool-seq data, with coordinates on TGU1A. Scores represent the maximum posterior probability of a selective sweep for each window in log scale (-log10(1-Prob)). (Abbreviations: inf, infinity)

File Name: Supplementary Data 2
Description: Sample information for the three bill morphologies of adult P. ostrinus birds originating from Cameroon. Asterisks (*) indicate individuals were resequenced. (Abbreviation: lower mandible width in mm, LMW)
