# Supplementary material for: Growth factor gene IGF1 is associated with bill size in the black-bellied seedcracker Pyrenestes ostrinus
Source: Nat Commun. 2018 Nov 19;9:4855. doi: 10.1038/s41467-018-07374-9 (PMC6242981; doi:10.1038/s41467-018-07374-9)
Supplement: Supplementary file 5 — Reporting Summary [file 41467_2018_7374_MOESM5_ESM.pdf]

nature research

Corresponding author(s): *Double-blind peer review submissions: write DBPR and your manuscript number here instead of author names.*

Reporting Summary

Nature Research wishes to improve the reproducibility of the work that we publish. This form provides structure for consistency and transparency in reporting. For further information on Nature Research policies, see Authors & Referees and the Editorial Policy Checklist.

Statistical parameters

When statistical analyses are reported, confirm that the following items are present in the relevant location (e.g. figure legend, table legend, main text, or Methods section).

n/a	Confirmed	
☐	☒	The exact sample size (*n*) for each experimental group/condition, given as a discrete number and unit of measurement
☐	☒	An indication of whether measurements were taken from distinct samples or whether the same sample was measured repeatedly
☐	☒	The statistical test(s) used AND whether they are one- or two-sided<br>*Only common tests should be described solely by name; describe more complex techniques in the Methods section.*
☐	☒	A description of all covariates tested
☐	☒	A description of any assumptions or corrections, such as tests of normality and adjustment for multiple comparisons
☐	☒	A full description of the statistics including central tendency (e.g. means) or other basic estimates (e.g. regression coefficient) AND variation (e.g. standard deviation) or associated estimates of uncertainty (e.g. confidence intervals)
☐	☒	For null hypothesis testing, the test statistic (e.g. *F*, *t*, *r*) with confidence intervals, effect sizes, degrees of freedom and *P* value noted<br>*Give P values as exact values whenever suitable.*
☐	☒	For Bayesian analysis, information on the choice of priors and Markov chain Monte Carlo settings
☒	☐	For hierarchical and complex designs, identification of the appropriate level for tests and full reporting of outcomes
☒	☐	Estimates of effect sizes (e.g. Cohen's *d*, Pearson's *r*), indicating how they were calculated
☐	☒	Clearly defined error bars<br>*State explicitly what error bars represent (e.g. SD, SE, CI)*

Our web collection on statistics for biologists may be useful.

Software and code

Policy information about availability of computer code

Data collection	We used some sequence processing functions in CLC Genomics Workbench, a proprietary software with references to peer-reviewed literature and methods. The remainder of the data processing is freely available.
Data analysis	The analytical software used is freely and publicly available. We did not use any custom code.

For manuscripts utilizing custom algorithms or software that are central to the research but not yet described in published literature, software must be made available to editors/reviewers upon request. We strongly encourage code deposition in a community repository (e.g. GitHub). See the Nature Research guidelines for submitting code & software for further information.

Data

Policy information about availability of data

All manuscripts must include a data availability statement. This statement should provide the following information, where applicable:

- Accession codes, unique identifiers, or web links for publicly available datasets
- A list of figures that have associated raw data
- A description of any restrictions on data availability

Demultiplexed and processed (i.e. trimmed and clipped) FASTQ files are available on NCBI SRA (accession SRP140635 [https://www.ncbi.nlm.nih.gov/sra/

SRP140635]) for the three pools used in Pool-seq (small-, large-, and mega-billed morphs) and for the targeted capture resequencing of 12 small- and 12 large-billed individuals. We further deposited their respective BAM files, mapped to the reference taeGut2 genome.

Field-specific reporting

Please select the best fit for your research. If you are not sure, read the appropriate sections before making your selection.

☐ Life sciences ☐ Behavioural & social sciences ☒ Ecological, evolutionary & environmental sciences

For a reference copy of the document with all sections, see nature.com/authors/policies/ReportingSummary-flat.pdf

Ecological, evolutionary & environmental sciences study design

All studies must disclose on these points even when the disclosure is negative.

Study description	We explored the genetic associations to map the genomic region that influences bill size in an African finch. We first identify a single genomic location through pooled sequencing efforts, followed up with targeted resequencing and long-read sequence data collection. Our initial findings suggest a single haplotype is associated with increasing bill size and possibly suggestive of an inversion. However, after long-read sequencing, we do not find support for an inverted genetic element. We conduct association tests and report significance and other metrics.
Research sample	We utilized a curated collection of blood samples from Pyrenestes ostrinus, an African finch that has a bill size polymorphism with known connections to feeding ecology fitness and Mendelian inheritance.
Sampling strategy	Balancing a budget and samples available with meta-data, we initiated our genetic screen with a larger sampling and then were limited by reagent kit size and budget.
Data collection	Samples were prepared by the laboratory of a single coauthor and sequenced at a Genomics Core Facility.
Timing and spatial scale	The sampled all derive from juvenile or adult birds, when the morphological trait is well established and stabilized. The year or time at which samples were collected have no impact on the genetics associated with the individual trait.
Data exclusions	NA
Reproducibility	There was a larger panel of samples used to discover the genomic regions of interest, with a subset selected for mapping.
Randomization	We utilize the beak morphology and the population of origin. We conduct genetic structure analyses to determine the role of population structure.
Blinding	We required the knowledge of the phenotype class in order to explore the genetic variation associated with each class.

Did the study involve field work? ☐ Yes ☒ No

Reporting for specific materials, systems and methods

Materials & experimental systems

n/a	Involved in the study
☐	☒ Unique biological materials
☒	☐ Antibodies
☒	☐ Eukaryotic cell lines
☒	☐ Palaeontology
☒	☐ Animals and other organisms
☒	☐ Human research participants

Methods

n/a	Involved in the study
☒	☐ ChIP-seq
☒	☐ Flow cytometry
☒	☐ MRI-based neuroimaging

Unique biological materials

Policy information about availability of materials

Obtaining unique materials	The senior author maintains a collection of whole blood from the species of interest. It is not common to possess this specific species, hence our consideration of them as "unique".
